# The recognition of structured elements by a conserved groove distant from domains associated with catalysis is an essential determinant of RNase E

**DOI:** 10.1093/nar/gkac1228

**Published:** 2023-01-03

**Authors:** Justin E Clarke, Kiran Sabharwal, Louise Kime, Kenneth J McDowall

**Affiliations:** Astbury Centre for Structural Molecular Biology, School of Molecular and Cellular Biology, Faculty of Biological Sciences, University of Leeds, Leeds, LS2 9JT, UK; Astbury Centre for Structural Molecular Biology, School of Molecular and Cellular Biology, Faculty of Biological Sciences, University of Leeds, Leeds, LS2 9JT, UK; Astbury Centre for Structural Molecular Biology, School of Molecular and Cellular Biology, Faculty of Biological Sciences, University of Leeds, Leeds, LS2 9JT, UK; Astbury Centre for Structural Molecular Biology, School of Molecular and Cellular Biology, Faculty of Biological Sciences, University of Leeds, Leeds, LS2 9JT, UK

## Abstract

RNase E is an endoribonuclease found in many bacteria, including important human pathogens. Within *Escherichia coli*, it has been shown to have a major role in both the maturation of all classes of RNA involved in translation and the initiation of mRNA degradation. Thus, knowledge of the major determinants of RNase E cleavage is central to our understanding and manipulation of bacterial gene expression. We show here that the binding of RNase E to structured RNA elements is crucial for the processing of tRNA, can activate catalysis and may be important in mRNA degradation. The recognition of structured elements by RNase E is mediated by a recently discovered groove that is distant from the domains associated with catalysis. The functioning of this groove is shown here to be essential for *E. coli* cell viability and may represent a key point of evolutionary divergence from the paralogous RNase G family, which we show lack amino acid residues conserved within the RNA-binding groove of members of the RNase E family. Overall, this work provides new insights into the recognition and cleavage of RNA by RNase E and provides further understanding of the basis of RNase E essentiality in *E. coli*.

## INTRODUCTION

The translation machinery is amongst the most abundant cellular components in all domains of life (1–3). In rapidly growing *Escherichia coli* ∼250 000–400 000 tRNAs deliver charged amino acids to 30 000–60 000 ribosomes in the translation of a dynamic pool of mRNA transcripts that at any moment number 1500–3000 (4,5). Under conditions of rapid growth, despite the RNA components of the translational machinery being relatively stable (6), the need to populate both daughter cells at each division dictates that the production of RNA components of the translation machinery is one of the major activities associated with cell growth. In *E. coli*, the tRNA genes are transcribed as either polycistronic or monocistronic transcripts, which undergo extensive processing on both the 5′ and 3′ side of the tRNAs to generate the mature forms that can be aminoacylated (7).

RNase E, an endoribonuclease that is found in many bacteria including important pathogens of humans and in some plant plastids (8), plays an important, if not essential, role in *E. coli* tRNA processing by separating the individual pre-tRNAs from polycistronic transcripts (9–12). Sites at which RNase E cleaves tRNA precursor have been mapped *in vivo* (10,11), many with nucleotide resolution (10,13), and preparations of RNase E have been shown to be capable of cleaving at such sites *in vitro* (13,14). This, along with tRNAs being structurally stable and well defined (15,16), makes these precursors suitable substrates for dissecting the molecular basis of the recognition and cleavage of RNA by RNase E (see Supplementary Figure S1).

RNase E also has a major role in the turnover of the greater part of the mRNA pool in *E. coli* (8,13,17). Much of the earlier work on RNase E focused on the ability of 5′-untranslated regions (5′-UTRs) to affect the overall rate of decay of mRNA transcripts (18). This led to the discovery that RNase E can interact with 5′-monophosphorylated ends (19,20), which can be converted from 5′-triphosphorylated ends (21) by routes that appear to be absolutely dependent on RppH, an RNA 5′ pyrophosphohydrolase (22,23). Whilst it has been established that the generation of 5′-monophosphorylated ends and their accessibility to RNase E can be determinants of RNA stability for some mRNAs, the rate of decay of many, if not most transcripts in cells lacking RppH is unaffected (22). This is further supported by the fact that RppH itself is non-essential in *E. coli* (22,24).

It has also been shown that RNase E can cleave many substrates rapidly independent of their 5′-phosphorylation status (13,14,25), and sites of RNase E cleavage have been mapped at distance from 5′-UTRs, e.g. within the 3′-UTRs of *cspA* and *rpsO* mRNA (26,27). The removal of Rho-independent transcription terminators greatly enhances degradation by 3′ to 5′ exonucleases (28). In the absence of translation, mRNAs are increasingly susceptible to RNase E cleavage internal to the protein-coding segment (29–32). Contrary to previous thinking (32), sites of efficient RNase E cleavage independent of access to a 5′-monophosphorylated end appear to be common rather than special cases.

The catalytic activity of *E. coli* RNase E is a function of its N-terminal half (NTH) (33), which forms a tetramer via the dimerization of dimeric units (34,35). Each dimeric unit contain two symmetrical active sites, each located within a DNase I-like domain, which along with an S1 domain can form a channel that binds single-stranded RNA. Located adjacent to each channel is a pocket called the 5′-sensor that can bind a 5′-monophosphorylated end (34). The recognition of 5′-monophosphorylated ends by the 5′-sensor can contribute to the overall stability of an interaction between RNase E and a substrate (36,37). The absence of a 5′-monophosphorylated end does not appear to present an intrinsic barrier to RNase E cleavage provided overall binding is supported by other interactions. In work leading to the present study, access to adjacent but not contiguous single-stranded regions has been shown to be a requirement for at least some RNase E cleavages within the context of tRNA maturation (13,14). Here we present evidence that a recently discovered RNA-binding groove (38) distant from the domains of RNase E associated with catalysis (34,39) also has a critical role in the processing of tRNA, is essential for the viability of *E. coli* and may represent a key point of evolutionary divergence from the paralogous RNase G family (40,41). The wider implications of our findings beyond the processing of tRNA precursors are discussed.

## MATERIALS AND METHODS

### Synthesis of RNA transcripts

Transcripts were synthesized *in vitro* using T7 RNA polymerase and polymerase chain reaction (PCR)-generated templates and purified, as described previously (13,14). The concentration and integrity of RNA samples were determined using a NanoPhotometer^®^ P-300 (Geneflow) and polyacrylamide gel electrophoresis (PAGE), respectively. The sequences of the primers used to generate cDNA templates are provided in Supplementary Table S1.

### Site-directed mutagenesis

Site-directed mutagenesis was performed using a QuikChange Multi Site-Directed Mutagenesis Kit according to the manufacturer's instructions (Agilent). Primers for mutagenesis were designed using the online QuikChange Primer Design tool (https://www.agilent.com/store/primerDesignProgram.jsp) and are provided in Supplementary Table S2. Transformations were performed with 2 μl of mutagenic reactions and 50 μl of chemically competent XL10-Gold cells (Agilent). Successful mutagenesis was confirmed by Sanger sequencing (GeneWiz).

### Purification of NTH-RNase E and discontinuous cleavage assays

Recombinant polypeptides corresponding to the NTH of *E. coli* RNase E (residues 1–529) were purified as described previously (34). All were tagged with oligohistidine at their N-terminus. Polypeptides with wild-type sequence, T170V substitution and D346N substitution have been described previously (35). New to this study was a polypeptide with eight substitutions (R3Q, Q22N, H268S, Y269F, Q270N, K433N, R488Q and R490Q) within a recently described RNA-binding groove, referred to as the 8x mutant herein (38). Discontinuous cleavage assays were performed in a buffer containing 25 mM *bis*-Tris propane (pH 8.3), 100 mM NaCl, 15 mM MgCl_2_, 0.1% (v/v) Triton X-100, 1 mM dithiothreitol (DTT) and 32 U of RNaseOUT™ ribonuclease inhibitor (Invitrogen). Reactions were started by combining enzyme (in reaction buffer) with RNA substrate, both of which had been pre-incubated separately at 37°C for 20 min. Aliquots were taken at each time point and quenched by adding to an equal volume of 2× RNA loading dye: 95% (v/v) formamide, 0.025% (w/v) bromophenol blue, 0.025% (w/v) xylene cyanol and 0.025% (w/v) sodium dodecylsulphate (SDS). The samples were analysed by denaturing PAGE and stained using ethidium bromide.

### Kinetic analyses

For the Michaelis–Menten analysis, reactions were performed under similar conditions to those above. Final substrate concentrations were: 0.5, 1, 2, 4, 6, 8 and 10 μM. The final enzyme concentration was 40 nM. Samples were separated and analysed by denaturing PAGE alongside a series of dilutions of the substrate at concentrations of 0, 6.25, 12.5, 25, 50, 100 and 500 nM. Initial rates of product formation were calculated from time points in the linear phase of the reaction using the calibration curve of the serial dilutions. These rates were then fitted by non-linear regression to the Michaelis–Menten equation as shown in Equation 1:

(1) \begin{eqnarray*}\frac{v}{{\left[ E \right]}} = \frac{{{k_{{\rm{cat}}}}\left[ S \right]}}{{{K_{\rm{M}}} + \left[ S \right]}}\end{eqnarray*}

where *v* represents the initial rate, [*E*] represents the total enzyme concentration, *k*_cat_ represents the enzyme turnover number, [*S*] represents the initial substrate concentration and *K*_M_ represents the Michaelis constant.

For the single-turnover experiment, final substrate and enzyme concentrations were 5 nM and 200 nM, respectively. Samples were taken at the following time points: 0, 5, 15, 30, 60 and 120 s, and separated by denaturing PAGE, as described above. Gels were stained with SYBR^®^Gold Nucleic Acid Stain (Thermofisher) and visualized on a Fujifilm FLA-5000 scanner with a Y510 filter and the excitation laser set to 478 nm.

### Circular dichroism

5′-hydroxylated LU13 (5′-GAGACAGU↓AUUUG; where the arrow corresponds to the RNase E cleavage site) and BR15 (5′-GGGGG_m_A_m_C_m_A_m_G_m_U_m_A_m_U_m_U_m_U_m_G; where mN corresponds to 2′-*O*-methylated ribonucleotides) were diluted to a concentration of 7.5 μM in 25 mM *bis*-Tris propane (pH 8.3), 100 mM KCl, 15 mM MgCl_2_, 0.1% (v/v) Triton X-100 in a final volume of 240 μl. Derivatives of these substrates have been described previously (25,42). The solutions were placed in a quartz cuvette with a path length of 1 mm. The cuvettes were placed into a Jasco J-715 spectropolarimeter and allowed to equilibrate to 37°C for 10 min. Two circular dichroism (CD) scans were performed at 50 nm/min over a range of 220–320 nm with a 2 s response time, 1 nm pitch and 1 nm bandwidth. The slit width was set to 1000 μm. The CD spectrum of the buffer was taken first and was subtracted from the average spectrum of each oligonucleotide. Molar ellipticity was then calculated using the near UV equation as shown in Equation 2:

(2) \begin{eqnarray*}\left[ \theta \right] = \frac{\theta }{{10cl}}\end{eqnarray*}

where [*θ*] represents the molar ellipticity, *θ* represents the ellipticity measured, *c* represents the molarity and *l* represents the path length of the cuvette. Data were then zero-corrected at 320 nm. High tension values remained below 450 mV for all experiments, indicating a high signal to noise ratio.

### Binding assays

Ligand binding assays (LBAs) consisted of a 2-fold dilution series of NTH-RNase E in 25 mM *bis-*Tris propane (pH 8.3), 100 mM KCl, 15 mM CaCl_2_, 0.1% (v/v) Triton X-100, 1 mM DTT and 20% (v/v) glycerol. Each reaction (final volume of 20 μl) contained final reporter concentrations of 20 nM for transcripts for LU13 or 7.5 nM for BR15. Reactions were incubated at 37°C for 20 min before separating in 1% (w/v) agarose gels in 1× TBE at 10 V/cm for 35 min. LBA gels consisting of transcripts as reporters were stained with SYBR^®^Gold Nucleic acid stain. Gels were visualized on the Fujifilm scanner as described above. Band intensities were quantified using ImageJ (43) and used to plot graphs of bound RNA (intensity of bands corresponding to bound species normalized against total band intensities) as a function of NTH-RNase E concentration. *K*_d_ values were calculated as the concentration of RNase E when 50% of the transcript is bound to the enzyme, as determined by plotting a logistics curve using OriginPro (44).

Competition binding assays (CBAs) consisted of a 2-fold dilution series of the competing transcript prepared in a similar manner to LBAs. Each reaction (final volume of 20 μl) contained final concentrations of BR15 (as quadruplex) and NTH-RNase E of 7.5 nM and 20 nM, respectively. The concentration of competitor RNA in the dilution series can be found in each figure legend. Assays were performed and analysed in a similar manner to LBAs. *IC*_50_ values were calculated as the concentration of competing RNA required to cause dissociation of 50% of the BR15 from the NTH.

### Cell viability assays

**Table 1.** Plasmids used in cell viability assays

Plasmid	Features	Source
pRNE1000	Gene encoding full-length wild-type RNase E under the control of a constitutive promoter	(45)
pRNE1008	As pRNE1000 with 8× mutations associated with the RNA-binding groove	This study. See Supplementary Table S2.
pNRNE1000	As pRNE1000 with a mutation introducing a stop codon after the codon encoding amino acid 529	This study. See Supplementary Table S2.
pNRNE1008	As pNRNE1000 with 8× mutations associated with the RNA-binding groove	This study. See Supplementary Table S2.
pMPM-k1	Empty vector control	(45)

Competent *E. coli* CJ1832 cells (46) were transformed with the plasmids shown in Table 1. Cultures were plated onto LB agar plates supplemented with 25 μg/ml kanamycin and 1 mM isopropyyl-β-d-thiogalactopyranoside (IPTG), and incubated overnight at 37°C. To assess colony-forming ability, three separate colonies of each strain were resuspended in 50 μl of Luria–Bertani (LB) broth and were streaked on LB agar plates supplemented either with kanamycin only or with kanamycin and IPTG, followed by incubation at 37°C overnight.

To assess growth in liquid medium, three separate colonies of each strain were resuspended in 500 μl of LB broth and 2 μl of that was used to inoculate 200 μl of LB broth supplemented with either kanamycin only or kanamycin and IPTG. Growth was monitored in a clear 96-well microtitre plate (Greiner Bio-One) by measuring the OD_600 nm_ of the culture using a FLUOstar Omega plate reader (BMG Labtech) with orbital shaking at 200 rpm between readings.

### Multiple-sequence alignment

The amino acid sequences of *E. coli* K12 RNase E (accession number P21513-1) and RNase G (accession number P0A9J0-1) were obtained from UniProtKB. Amino acid sequences of the closest RNase G homologue from *Xylella fastidiosa, Pseudomonas aeruginosa, Haemophilus influenzae, Pasteurella multocida, Vibrio cholera, Yersinia pestis* and *Salmonella enterica* were obtained by BLASTp analysis using the *E. coli* RNase G sequence. A multiple-sequence alignment of all RNase E/G amino acid sequences was performed using Clustal Omega. Data weres visualized using Jalview (version 2.11.2.2).

## RESULTS

### Cleavage of tRNA precursors by RNase E is dependent on the presence of an adjacent tRNA unit

**Figure 1.**
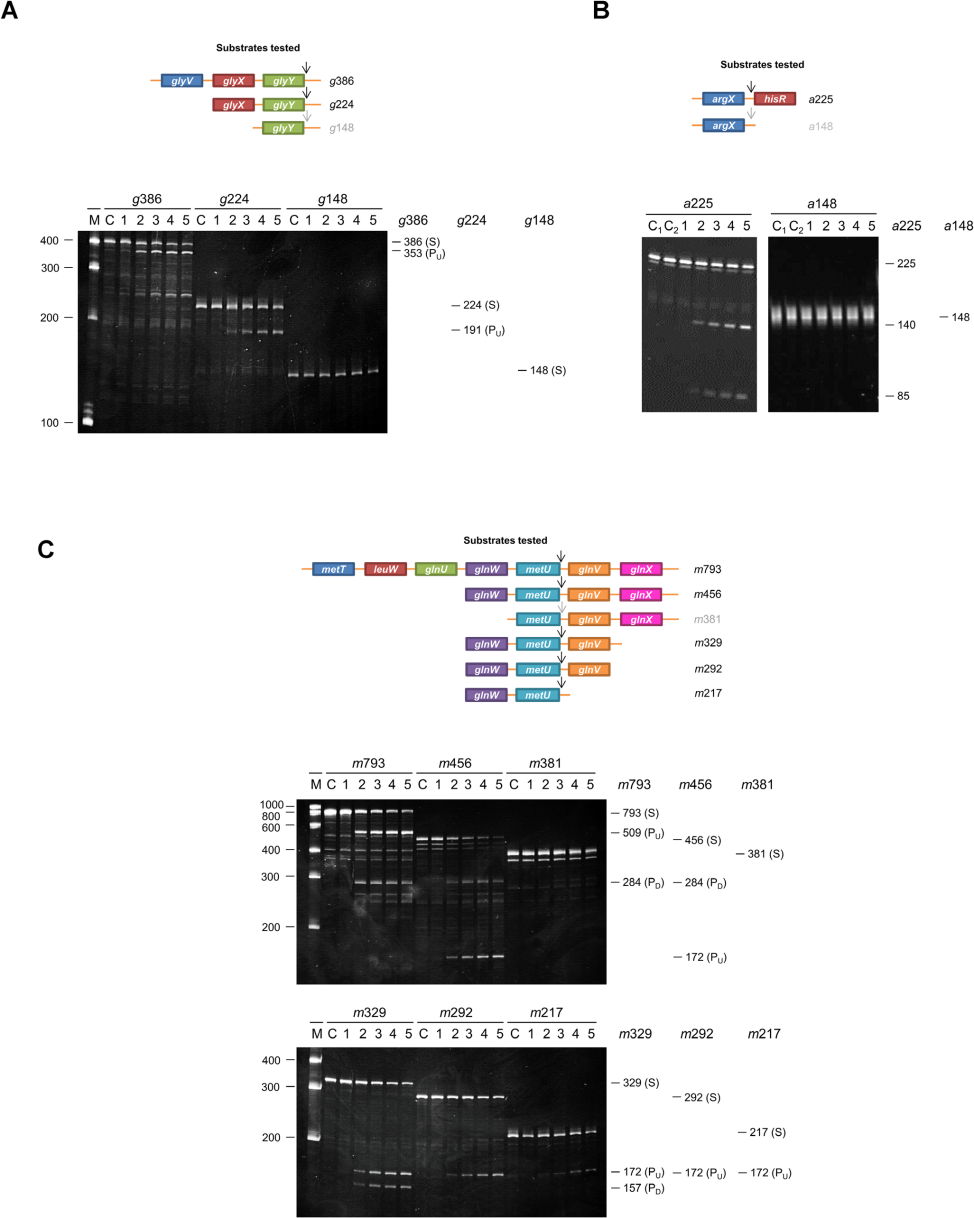
Identification of additional determinants of RNase E cleavage of tRNA precursors. (**A**) A schematic of the substrates used for analysis of cleavage of the *glyV–glyX–glyY* transcript (Supplementary Figure S1A). The tRNA units are labelled and coloured. The names of the substrates are indicated at the right, which are coloured black if these substrates were shown to be cleaved by RNase E or grey if they were not cleaved. The numbers that are incorporated into the names of the substrates indicate their size (nt). The sequences of oligonucleotides used to generate the templates for *in vitro* transcription are provided in Supplementary Table S1. Below this schematic are the cleavage assay results. Reactions were performed as described in the Materials and Methods using the NTH half of RNase E. Final substrate and enzyme concentrations were 180 and 20 nM, respectively. Lanes 1–5 contain samples taken after 0, 5, 15, 30 and 60 min of initiating the reaction. Lane C contains the substrate incubated without enzyme for 60 min. Lane M contains a RiboRuler™ low range RNA ladder (Invitrogen) with the lengths of selected fragments in nucleotides indicated at the left of each image. The lengths in nucleotides of the substrates and cleavage products are shown at the right of the image. The descriptors S, P_U_ and P_D_ highlight the bands representing the substrate, the upstream product and the downstream product of cleavage at the designated RNase E site. (**B**) A schematic of substrates derived from the *argX–hisR* segment of the *argX–hisR–leuT–proM* transcript (Supplementary Figure S1B), as well as the cleavage assay results for these substrates. Labelling and numbering are as in (A). Lanes C1 and C2 represent control reactions with substrate incubated without enzyme for 0 and 60 min, respectively. (**C**) A schematic of the *metT–leuW–glnU–glnW–metU–glnV–glnX* transcript (Supplementary Figure S1C), as well as the cleavage assay results for these substrates. Labelling and numbering are as in (A).

Previous analysis of the cleavage of tRNA precursors by RNase E revealed multiple examples where access to a single-stranded region on one side of a tRNA enhanced cleavage on the other, independently of interaction with a 5′-monophosphorylated end (13,14). However, further dissection of three tRNA precursors revealed an additional determinant of RNase E cleavage (Figure 1; see Supplementary Figure S1 for nascent substrates). Analysis of the *glyV*–*glyX*–*glyY* transcript (Figure 1A) revealed that efficient RNase E cleavage on the 3′ side of *glyY*-tRNA requires, in addition to an accessible single-stranded region on the 5′ side of *glyY*-tRNA (13), the upstream *glyX*-tRNA. The *glyV*-tRNA, the first of the tricistronic operon, does not appear to contribute to cleavage on the 3′ side of *glyY*-tRNA, suggesting that a tandem arrangement of tRNA may be sufficient. Analysis of the first half of the tetracistronic transcript *argX–hisR–leuT–proM* revealed that efficient RNase cleavage on the 3′ end of *argX*-tRNA, which lacks an upstream tRNA as *argX*-tRNA is the first tRNA, requires access not only to the 5′ leader (14) but also to *hisR*-tRNA, which is immediately downstream (Figure 1B). This result was shown within the context of a transcript truncated on the 3′ side of *hisR*-tRNA. In a manner similar to the RNase E cleavage 3′ to *glyY-*tRNA, cleavage on the 3′ side of *metU*-tRNA within the heptacistronic transcript *metT–leuW–glnU–glnW–metU–glnV–glnX* requires not only a single-stranded region on the 5′ side of *metU*-tRNA (13) but also the upstream *glnW*-tRNA (Figure 1C). The above experiments indicated that tRNAs adjacent to tRNAs bordered by single-stranded regions that are recognized by RNase E can be important determinants of cleavage efficiency.

### Adjacent tRNA units bind to RNase E at a site distant from catalysis

**Figure 2.**
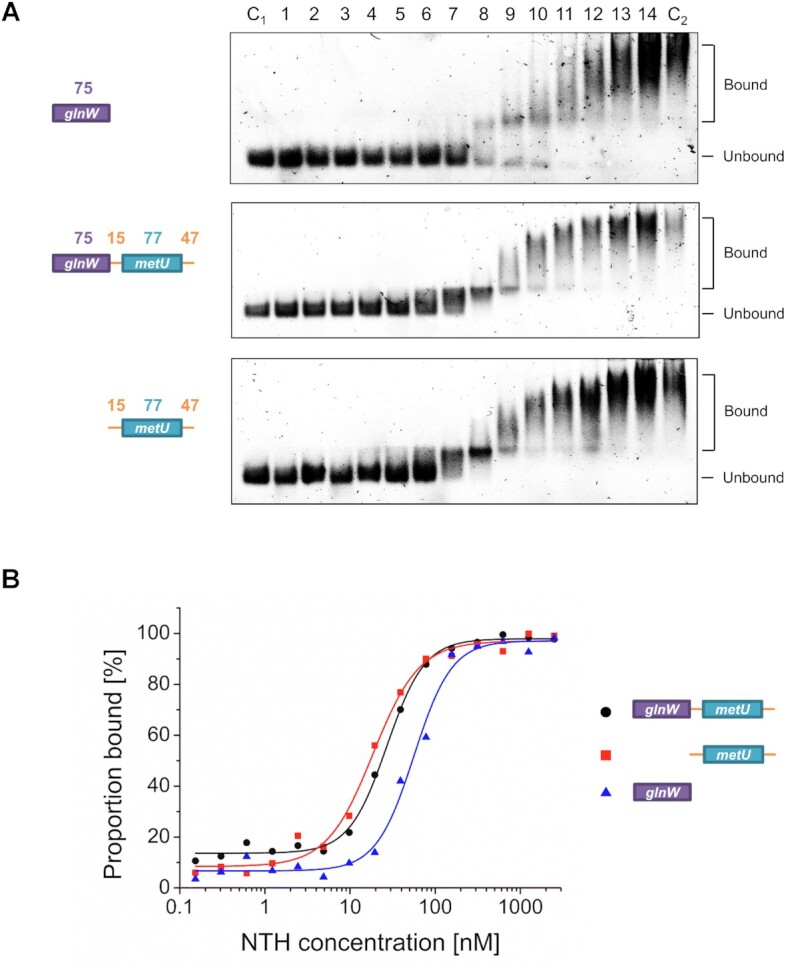
Binding of RNase E to fragments of the *metT* tRNA precursor. (**A**) Electrophoretic mobility shift assays (EMSAs) of *metT* RNA fragments incubated with increasing concentrations of NTH-RNase E with the D346N substitution. Lanes 1–14 contain 20 nM RNA fragments incubated with 0.3, 0.6, 1.2, 2.4, 4.9, 9.8, 19.5, 39, 78.1, 156.3, 312.5, 625 nM, and 1.25 and 2.5 μM NTH-RNase E D346N, respectively. Lanes C_1_ and C_2_ contain 20 nM RNA fragments and 2.5 μM NTH-RNase E D346N, respectively. The first, second and third images (from top to bottom) correspond to *glnW*-tRNA, the entire *glnW–metU* segment and *metU*-tRNA with 5′- and 3′-single-stranded regions, respectively. A schematic is provided for all three fragments at the left of the images, with numbering and labelling as described in Supplementary Figure S1. (**B**) Binding curves of the assays in panel A. The blue, black and red curves correspond to *glnW*-tRNA, the *glnW–metU* segment and *metU*-tRNA with 5′- and 3′-single-stranded regions, respectively. Schematics are provided as in (A).

The ability of RNase E to bind tRNA substrates was then investigated using a D346N mutant of NTH-RNase E, which shows a substantial reduction in catalytic activity as a consequence of the substitution of an aspartate required for coordination of the active-site magnesium ion (34). Thus, it was possible to study binding in the absence of detectable substrate cleavage. RNase E was able to bind *glnW*-tRNA that lacked single-stranded regions on both the 5′ and 3′ side (Figure 2A). The *K*_d_ value of 56 nM for this interaction was only 2-fold higher than the *K*_d_ value of 27 nM for the *glnW–metU* fragment, which includes single-stranded regions shown to contribute to RNase E cleavage (Figure 2B). The results suggested that tRNAs as well as flanking single-stranded regions contribute to RNase E binding. Consistent with this suggestion, the *K*_d_ value of 19 nM for *metU*-tRNA with flanking single-stranded regions was lower than the *K*_d_ value of 56 nM obtained for *glnW*-tRNA without flanking single-stranded regions (Figure 2B). The smearing toward the top of the gel at the highest concentrations of D346N (compare lanes 16 and C_2_) is caused by non-stoichiometric amounts of contaminating RNA in NTH-RNase E preparations (estimated to be <0.01% by mass). The affinities of the interactions assayed above are similar to that of RNase E with 5′-monophosphorylated LU13 (*K*_d_ value of 56 nM, Supplementary Figure S2), a synthetic reference substrate (25,42,47,48) known to be cleaved rapidly by wild-type RNase E via interaction with the 5′-sensor pocket and the channel that binds single-stranded RNA (see the Introduction). In the absence of the 5′-monophosphate group (i.e. when only a single single-stranded region is present), the affinity of RNase E for LU13 decreases by at least 50-fold (*K_d_* of >10 μM; Supplementary Figure S2). These binding results for LU13 are consistent with previous work that showed that a 5′-monophosphate group does not enhance the turnover number of RNase E and instead decreases the *K*_M_ (14).

To determine whether binding of *glnW*-tRNA to RNase E involved regions of the protein also involved in the recognition of single-stranded regions of RNA, we used a competition-binding assay (CBA) with another synthetic reference substrate BR15. This substrate presents four single-stranded regions through the formation of a stable G-quadruplex at the 5′ end (25), which was confirmed by CD (Supplementary Figure S3). Prior to being used in the CBA, it was confirmed that the interaction of BR15 and NTH-RNase E has a relatively high affinity (*K_d_* of 7.8 nM) and is susceptible to competition by a substrate that presents a single single-stranded region provided the 5′ end was monophosphorylated (Supplementary Figure S4).

**Figure 3.**
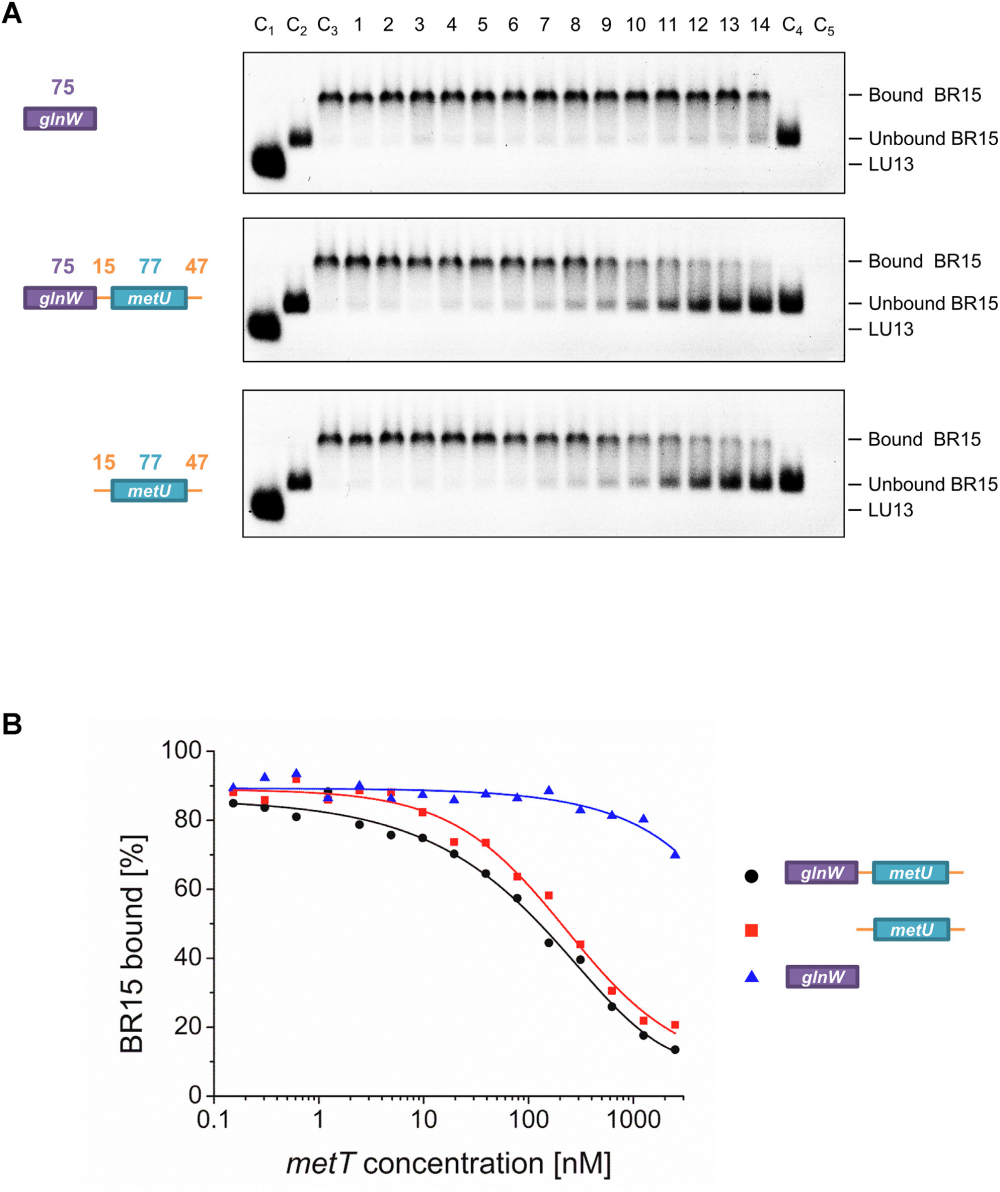
Assaying the requirement for interaction with sites that bind single-stranded segments. (**A**) The result of competition assays between the *glnW–metU* segment and subfragments. Lanes 1–14 contain 7.5 nM labelled BR15 (quadruplex) and 20 nM NTH-RNase E incubated with 0.3, 0.6, 1.2, 2.4, 4.9, 9.8, 19.5, 39, 78.1, 156.3, 312.5, 625 nM, and 1.25 and 2.5 μM competing transcript. Lanes C_1_, C_2_, C_3_ and C_4_ contain 30 nM LU13 with a 3′-fluorescein label, 7.5 nM BR15, 7.5 nM BR15 incubated with 20 nM wild-type NTH-RNase E and 20 nM NTH-RNase E incubated with 2.5 μM unlabelled competing transcript. The expected binding states of each band are shown at the right of each image. The first, second and third images (from top to bottom) correspond to competition with a fragment containing *glnW*-tRNA without a 5′- or 3′-single-stranded extension, the entire *glnW–metU* segment and the *metU*-tRNA flanked at both its 5′ and 3′ ends by single-stranded regions, respectively. A schematic is provided for all three fragments at the left of the images, with numbering and labelling as described in Supplementary Figure S1. (**B**) Binding curves of the assays in panel A. The blue, black and red curves correspond to *glnW*-tRNA, the *glnW–metU* segment and *metU*-tRNA with 5′- and 3′-single-stranded regions, respectively. Schematics are provided as in (A).

The *glnW–metU* segment and the subfragment that contained *metU*-tRNA flanked by single-stranded regions, but not *glnW*-tRNA without flanking single-stranded regions, were able to compete effectively with BR15 for binding (Figure 3). The *IC*_50_ values for the *glnW–metU* segment and the subfragment that contained *metU*-tRNA flanked by single-stranded regions were similar (229 nM and 147 nM, respectively), whilst the *IC*_50_ value for *glnW*-tRNA was at least a magnitude higher (>2.5 μM). The higher *IC*_50_ value for *glnW*-tRNA (compared with the values of the other two substrates) could not be accounted for by the smaller differences in *K_d_* values (Figure 2). Considered together, the above results suggested that RNase E binds to *glnW*-tRNA using a site that does not overlap functionally with the channel that binds single-stranded regions.

### Adjacent tRNA units contribute to cleavage by RNase E via an allosteric mechanism and can be substituted by simple stem–loops in mRNA

**Figure 4.**
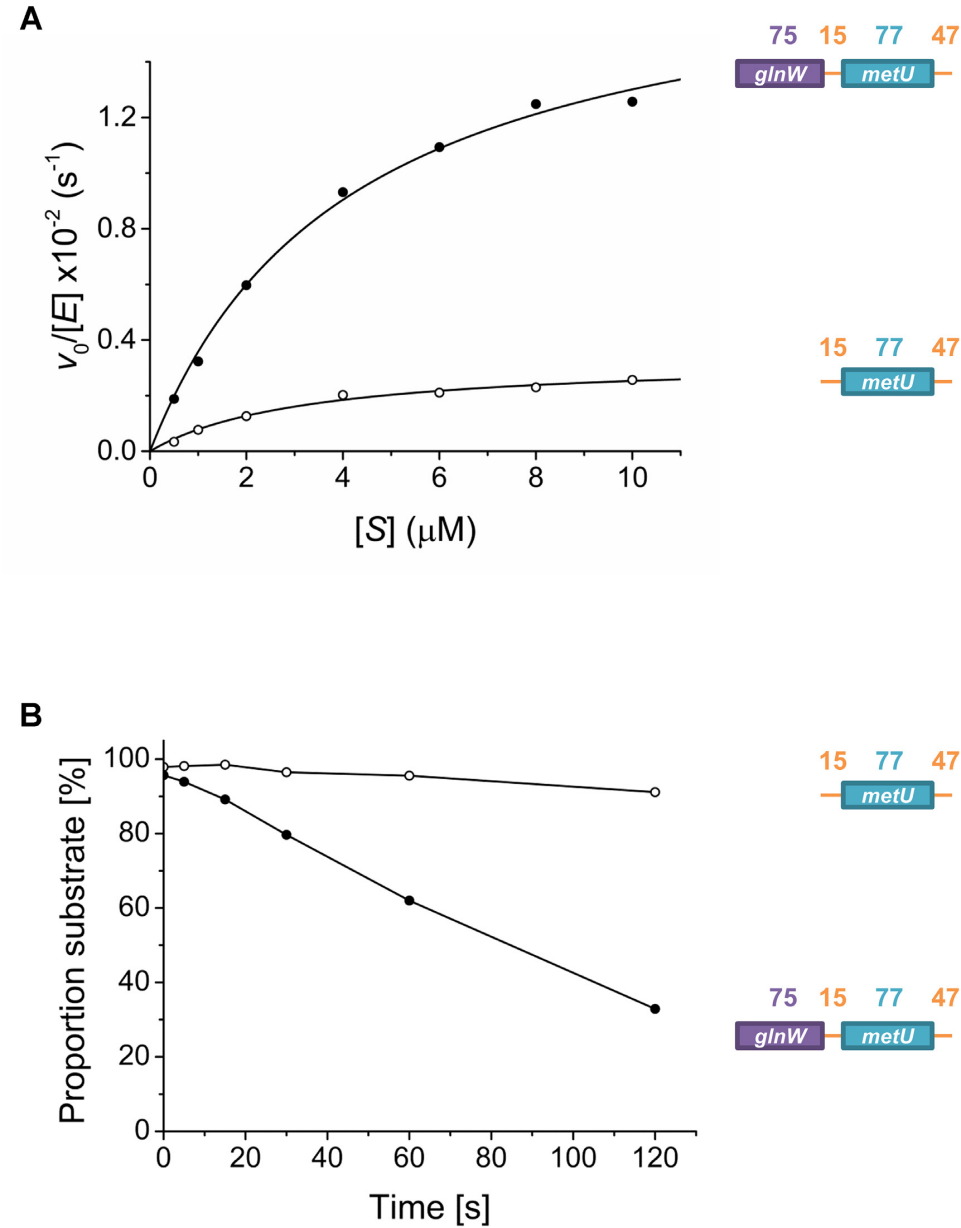
Analysing the influence of *glnW*-tRNA on cleavage downstream of *metU*-tRNA. (**A**) A Michaelis–Menten plot for RNase E cleavage downstream of *metU*-tRNA for the fragment containing *metU*-tRNA flanked at both its 5′ and 3′ ends by single-stranded regions, and the entire *glnW–metU* fragment, as shown by open and filled circles, respectively. The concentration of NTH-RNase E (monomer) in each reaction was 40 nM. Rates normalized against enzyme concentration (*v*_0_/[*E*]) were calculated as described previously (14,25,50), plotted against substrate concentration ([*S*]), and were fitted to the Michaelis–Menten equation. A schematic is provided for both fragments at the left of the images, with numbering and labelling as described in Supplementary Figure S1. (**B**) A graph of the proportion of substrate over time following incubation with RNase E under single-turnover conditions. The concentration of substrate and NTH-RNase E (monomer) in each reaction was 5 nM and 200 nM, respectively. The colouring is the same as for (A). Schematics are provided as in (A).

To explore further the contribution of *glnW*-tRNA, we undertook a Michaelis–Menten analysis of RNase E cleavage of *metU*-tRNA flanked at both its 5′ and 3′ ends by single-stranded regions and the larger *glnW–metU* fragment, which also includes *glnW*-tRNA (Figure 4A). *K*_M_ values of 3.2 and 3.7 μM were obtained for these substrates, respectively, which is consistent with both having similar *K*_d_ and *IC*_50_ values (Figures 2 and 3, respectively). The corresponding *k*_cat_ values were 3.35 × 10^−3^/s and 1.85 × 10^−2^/s, respectively. The 5.5-fold difference in these values suggested that the interaction with *glnW*-tRNA at a site not overlapping with the site of catalysis (as shown in Figure 3) is allosteric and increases catalytic turnover. Moreover, under conditions of enzyme excess, which minimize any effect of differences in product release (49), the rate of cleavage 3′ to *metU*-tRNA was 12-fold faster when *glnW* was present upstream (Figure 4B). The cleavage rates in the absence and presence of *glnW*-tRNA upstream were 1.16 × 10^−5^/s and 1.43 × 10^−4^/s, respectively. This suggests that the presence of *glnW*-tRNA may reduce the rate of product release and the level of allosteric activation may exceed that indicated by Michaelis–Menten analysis (Figure 4A).

**Figure 5.**
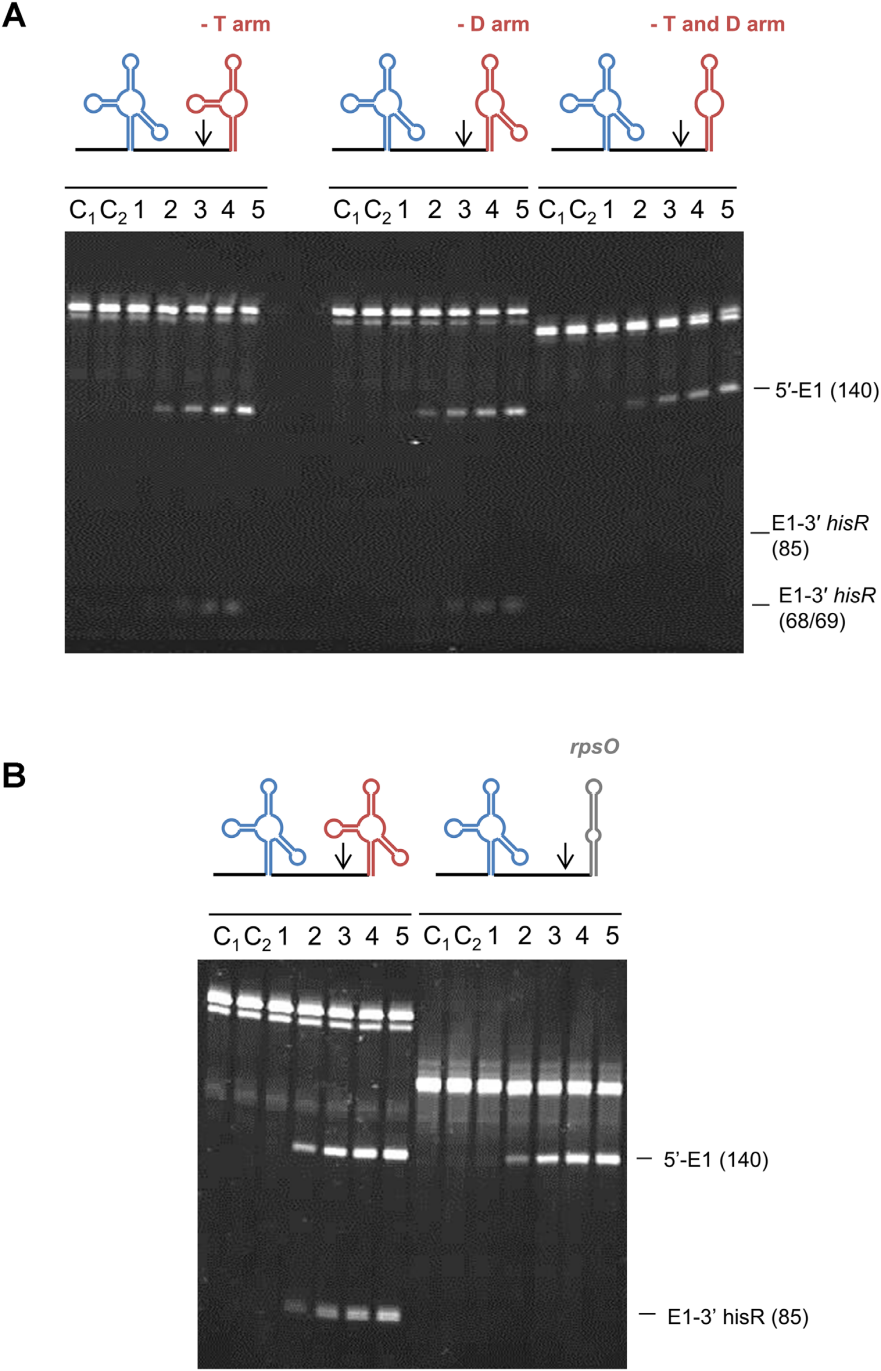
Investigating the contribution of *hisR*-tRNA to cleavage downstream of *argX*-tRNA. Schematics of the substrates are provided at the top of each gel. Vertical arrows show the position of an RNase E site that is not crucially dependent on 5′ sensing. Reactions were performed using the NTH of RNase E. Final substrate and enzyme concentrations were 180 and 5 nM, respectively. Lanes 1–5 contain samples taken after 0, 5, 15, 30 and 60 min of initiating the reaction. Lanes C_1_ and C_2_ contain substrate incubated without enzyme for 0 and 60 min, respectively. The sizes of the substrates and cleavage products are shown at the right of the image. (**A**) The cleavage assays of the entire *argX–hisR* fragment with dissections of the *hisR*-tRNA. (**B**) The cleavage assay of the *argX–hisR* fragment with replacement of the *hisR-*tRNA with the *rpsO* Rho-independent transcriptional terminator.

In order to explore the substrate requirements of tRNAs in binding to and allosterically activating RNase E, modified fragments of tRNA precursors were generated and subjected to further cleavage assays (Figure 5). Removal of the T or D arm or both of these arms, of *hisR-*tRNA on the *argX–hisR* fragment did not affect the ability of RNase E to cleave at its site downstream of *argX* (Figure 5A), despite the fact that removal of the entire *hisR-*tRNA does abolish cleavage (Figure 1B). Given that removal of both arms of the tRNA could potentially form an RNA secondary structure that was more similar to a basic stem–loop, a further substrate was generated that replaced the entire *hisR-*tRNA with the Rho-independent transcriptional terminator of the *rpsO* transcript. Cleavage of the RNA fragment downstream of the *argX*-tRNA even when the adjacent *hisR*-tRNA was replaced with the *rpsO* terminator was still prominent (Figure 5B), suggesting that the allosteric site on RNase E can interact with relatively simple stem–loops.

### The allosteric site is associated with a recently discovered RNA-binding groove, which is essential for cleavage of long RNA substrates and cell viability in *E. coli*

**Figure 6.**
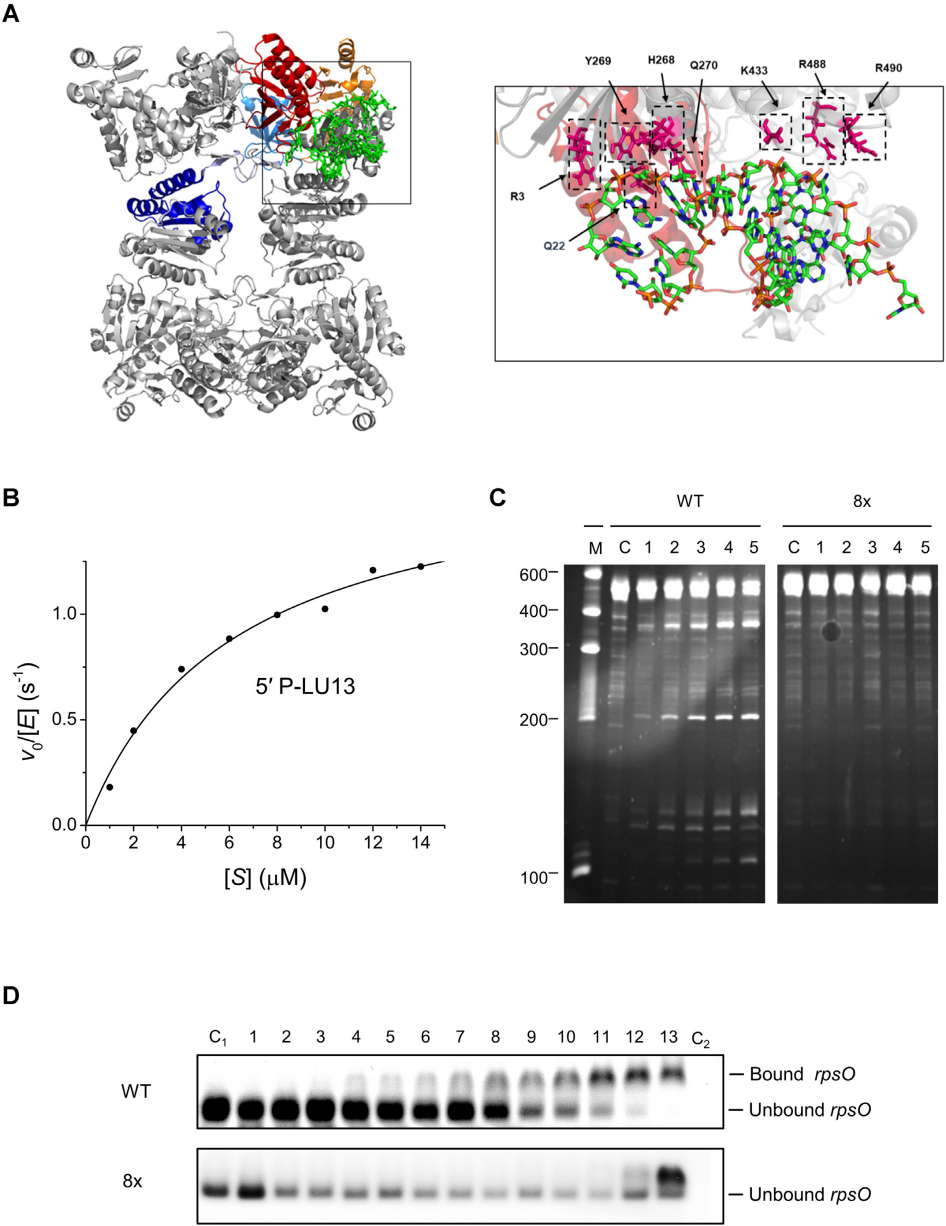
Substitutions within the groove that interacts with structured elements and their effects on tRNA binding. (**A**) The nature of the amino acid substitutions (R3Q, Q22N, H268S, Y269F, Q270N, K433N, R488Q, R490Q; this mutant is referred to as the 8x mutant herein) in the duplex-binding groove and their location relative to domains within the NTH of RNase E (PDB: 6G63). The domains coloured light grey, cyan, yellow, red and blue represent the RNase H-like domain, S1 subdomain, 5′-sensing domain, DNase I-like domain and the small domain, respectively. (**B**) A Michaelis–Menten plot for the cleavage of 5′-monophosphorylated LU13 by the RNase E 8× mutant. (**C**) Cleavage assays of *argX–hisR–leuT–proM* RNA in the presence of wild-type or mutant (8×) RNase E. Conditions were as described in Figure 1. (**D**) EMSAs of an RNA fragment consisting of the Rho-independent transcriptional terminator of *rpsO* incubated with increasing concentrations of NTH-RNase E. Lanes 1–13 contain 20 nM RNA fragments incubated with 1.2, 2.4, 4.9, 9.8, 19.5, 39, 78, 156.3, 312.5, 625, 1250, 2500 and 5000 nM NTH-RNase E, respectively. Lanes C_1_ and C_2_ contain only 20 nM RNA fragments and only 5000 nM NTH-RNase E, respectively. The top and bottom panel correspond to assays using wild-type or mutant (8×) RNase E, respectively.

In parallel with the functional studies presented here, others had identified through X-ray crystallographic studies a groove on RNase E that binds stem–loops within small regulatory RNAs (38). To determine if this groove binds tRNA, a variant of RNase E (termed the 8x mutant) was generated by introducing eight amino acid substitutions simultaneously along the length of this groove (R3Q, Q22N, H268S, Y269F, Q270N, K433N, R488Q and R490Q), which is non-overlapping with the sites of catalysis and the 5′-sensor, and encompasses the RNase H and small domain (Figure 6A). These substitutions did not affect the activity of the protein for 5′-monophosphorylated substrates, as observed using a Michaelis–Menten analysis with the substrate 5′-monophosphorylated LU13 (Figure 6B). *K*_M_ and *k*_cat_ values of 6.28 μM and 1.93/s, respectively, were obtained for the mutant with the eight substitutions in the groove. These values are similar to those reported for wild-type RNase E (14,25,50). In contrast, mutation of residues within the groove resulted in complete abolishment of cleavage of the *argX–hisR–leuT–proM* precursor (Figure 6C). The same groove substitutions also reduced the affinity of RNase E for the *rpsO* stem–loop (Figure 6D). Thus, the groove can bind a range of structured RNA segments and is likely to be a site of allosteric regulation of RNase E cleavage.

**Figure 7.**
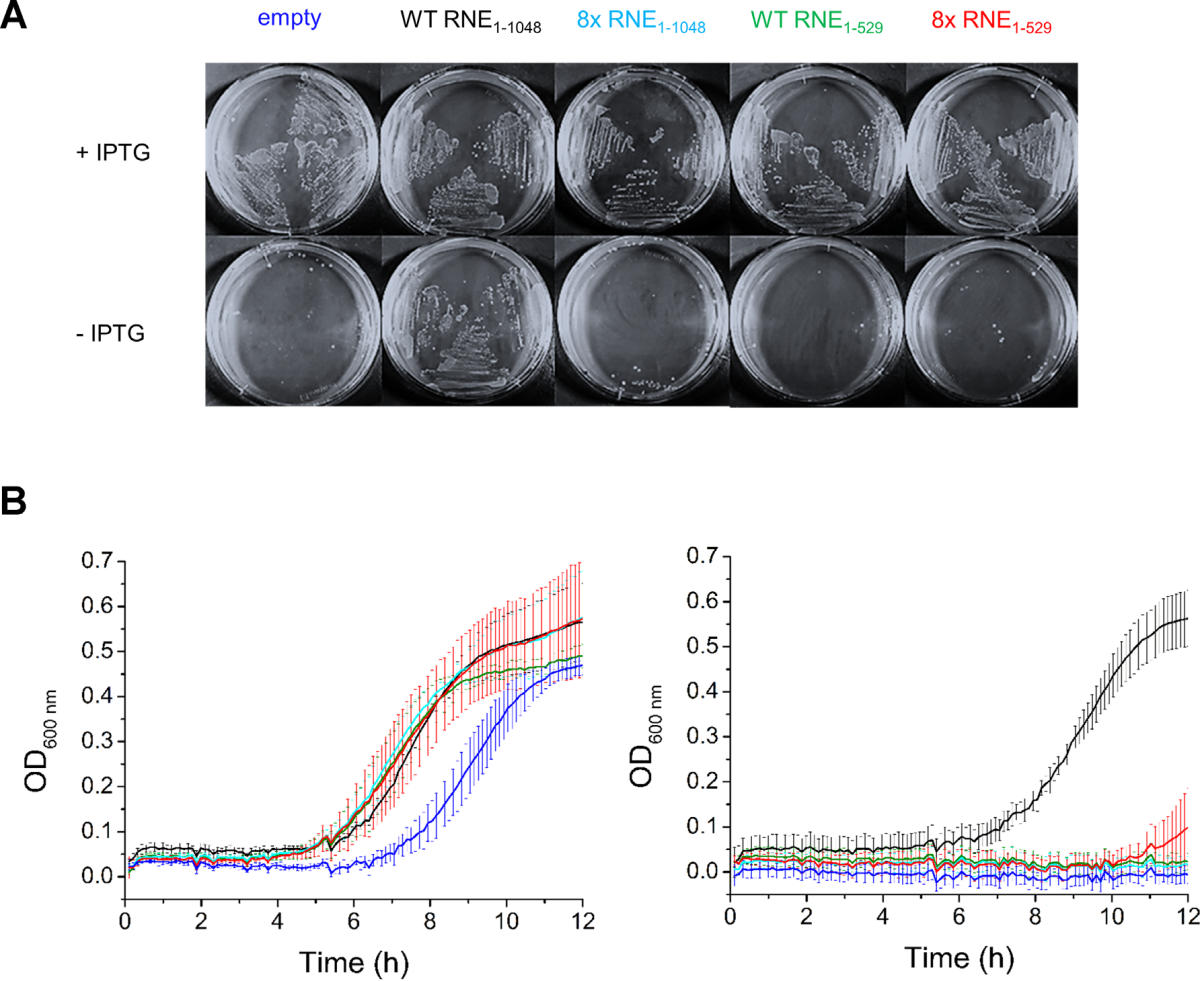
Complementation of RNase E deficiency to assess cell viability. CJ1832 strains contained the pRNE1000 plasmid encoding either full-length or NTH RNase E with the wild-type sequence or 8× substitutions. Full-length wild-type is indicated as black, full length 8× mutant as cyan, NTH wild-type as dark green and NTH 8× as red. CJ1832 containing the empty pMPM-k1 vector was included as a control and is shown as dark blue. (**A**) LB agar plates streaked with three separate colonies of the indicated strains with or without IPTG supplementation (1 mM). (**B**) Growth curves from liquid culture measured at OD_600 nm_ with (left) or without (right) IPTG supplementation. Three separate replicates were used for growth curves and the average curve is displayed with the standard error shown as solid lines. Western blots were performed to confirm similar expression levels between all the constructs (data not shown).

The finding that the substitutions within the groove had a substantial effect on the cleavage tRNA precursors predicted that they would also affect the growth and possibly the viability of *E. coli* given that RNase E cleavage activity is essential (24,48,51). This was tested in the context of full-length RNase E and an established system (45,46) to avoid the need to account for growth effects caused by the truncation of RNase E (48,52,53) and to allow direct comparison with previously published work (45,52). Mutations in the RNA-binding groove were introduced into the plasmid pRNE1000, which encodes the full-length RNase E under the control of the constitutive IS10 promoter and without autoregulation (45). To provide a control for reduced cell viability, site-directed mutagenesis was also used to introduce a stop codon after the codon encoding amino acid 529 in a wild-type sequence. The corresponding plasmids were introduced into the *E. coli* strain CJ1832 (46), which contains a chromosomal copy of RNase E that has been placed under the control of the *lac* promoter. Growth was detected on all plates in the presence of IPTG, indicating that there was no dominant-negative phenotype associated with the presence of the mutants. Strains harbouring the wild-type sequence, but not the sequence containing substitutions in the groove, were able to grow in the absence of IPTG (Figure 7A), indicating that the functional groove is essential for growth. As expected, the NTH of RNase E was insufficient to support normal growth within this system. The C-terminal half of RNase E has many important functions including sites of interaction with other proteins that drive assembly of the RNA degradosome complex (52,53). Similar results were found using liquid cultures (Figure 7B).

The RNA-binding groove may represent a key point of evolutionary divergence of RNase E from its paralogue RNase G, which does share sequence and structural homology but has limited functional overlap (41,54–56). Previously it has been shown that many residues of the groove found in *E. coli* RNase E were conserved in the orthologues of other bacterial species (38). However, an expanded sequence alignment revealed little to no conservation of these residues between *E. coli* RNase E and RNase G (Supplementary Figure S5). RNase G homologues found in various γ-proteobacteria were found to have conserved residues at these positions of a chemical nature less likely to make strong contacts with RNA. The arginine at position 3 in RNase E is conserved as an aspartate in RNase G orthologues, which reverses the charge at this position and could potentially disrupt ionic interactions with the RNA phosphodiester backbone. The glutamine at position 22 in RNase E is conserved as a glycine in RNase G, which would eliminate a potential hydrogen bond between the R group of the glutamine and the RNA. The histidine at position 268 in RNase E is present as leucine, valine or methionine in RNase G, which could all eliminate the ability to form hydrogen bonds with RNA. The tyrosine as position 269 seems to be conserved between RNase E and RNase G. The glutamine as position 270 in RNase E is present as either a glycine or an aspartate in RNase G. The residue lysine at position 433 in RNase E is mostly conserved as a leucine or alanine in RNase G. The arginine at position 488 in RNase E is mostly conserved as a leucine, valine or alanine in RNase G. The arginine at position 490 in RNase E is not present in RNase G, as all the peptide sequences for the orthologues shown here end before this amino acid. The last three residues associated with the RNA-binding groove of RNase E are similarly missing from RNase G and would be predicted to form strong ionic contacts with the phosphodiester backbone of the RNA. In contrast, other residues that are highly conserved in RNase E homologues, such as those involved in 5′-monophosphate interactions (R169 and T170) (34,39,48,57), the active site (F57, F67, D303 and D346) (34,57) and Zn link formation (C404 and C407) (34,58) are also found in RNase G and are conserved between RNase G homologues in other bacteria. Given that RNase G does not appear to be involved in the processing of tRNA or cleavage of many mRNA substrates (13,54,56) and is not essential for cell viability (56,59), these findings suggest that the RNA-binding groove may be a major feature that distinguishes RNase E and RNase G both functionally and phylogenetically.

## DISCUSSION

It has been shown here that the binding of tRNAs via a recently discovered groove in the N-terminal catalytic half of *E. coli* RNase E is a major determinant of the rate of processing of tRNA precursors (Figures 1–7). Moreover, the binding of structured elements is likely to be a widespread determinant of cleavage by *E. coli* RNase E. The groove that binds structured elements was located through co-crystallization of RNase E with small regulatory RNAs (38) and has been implicated in the generation of the 3′-UTR-derived small RNA MicL (60). Interaction with structured regions, which has long been implicated in the recognition and cleavage of RNA by RNase E (61–64), alongside interaction with single-stranded regions and 5′-monophosphorylated ends (13,14,19,25,36,39,42), may allow RNase E to recognize, cleave and remain associated with substrates via multiple and changing combinations of cooperative interactions in many aspects of RNA biology.

Interaction with the recently discovered groove, which is at distance from the site of catalysis, appears to stimulate RNA cleavage by an allosteric mechanism (Figures 2–4). It is interesting to note that a kink of ∼40° within the dimer–dimer interface is one of the major differences in the quaternary structure of the RNase E tetramer in the ‘open’ apoprotein form (39) compared with a ‘closed’ holoprotein form in which 5′-monophosphorylated oligonucleotides are bound via the 5′-sensor and the composite single-stranded RNA-binding channel formed by the DNase I and S1 domain (34). Each side of the dimer–dimer interface is composed of a pair of ‘small’ domains linked via chelation of a shared zinc (58). We suggest as a working model that interaction of RNA with the groove, which is a composite of surfaces from the small domain and the RNase H domain in the remainder of the NTH of RNase E, may reduce the kink, thereby promoting the adoption of the ‘closed’ confirmation, which in turn would enhance cleavage. What is being proposed is essentially a modified ‘mouse-trap’ model (34) in which conformational closing can be promoted by RNA interacting with the groove.

The recent study of the processing of the 3′-UTR-derived small RNA MicL has found a requirement for two stem–loops 3′ to the site of cleavage (60). Thus, as found here for tRNA [Figures 1 and 4; see also (14)], a tandem arrangement of structured elements may be a requirement for efficient RNase E cleavage at many sites including those at a distance from native 5′ ends. There is now no need to invoke models in which efficient cleavage within intergenic regions (13,14,25), 3′-UTRs (25–27,65) and other similarly distant sites (13,66,67) requires RNase E to remain tethered to a 5′-monophosphorylated end (8) with the intervening RNA being looped out (27). It seems more likely that within these regions structured segments in combination with single-stranded regions (13,14,25,26) contributed to ‘direct entry’ (25,26,32). The finding that RNase E can recognize a transcriptional terminator as part of an interaction that produces a cleavage upstream of the terminator (Figures 5 and 6) suggests that RNase E may have a widespread role in initiating 3′ to 5′ degradation. It is well established that transcriptional terminators impede the progress of 3′ to 5′ exonucleases (68–71).

Given the considerable capacity to bind RNA via a large number of combinations of individual sites, the question arises as to why the normal rates of decay of some transcripts are dependent on the RppH RNA pyrophosphohydrolase (22). The answer may simply be that in these but not all cases an interaction with a 5′-monophosphorylated end makes a substantial, non-replaceable contribution to RNase E cleavage. The close coupling of translation and transcription in *E. coli* and at least some other bacteria (72) means that much of the length of mRNAs will be protected by ribosomes (29,30,32), limiting the contacts initially available to 5′-UTRs. This is not to say that all mRNA degradation initiated at the 5′ end may require the generation of a 5′-monophosphorylated group: RNase E has been mapped in *E. coli* cells to 5′-UTRs of many mRNAs (30,31,73,74) and binds to a number of 5′-triphosphorylated UTRs *in vitro* (our unpublished data).

The study of tRNA processing *in vivo* has revealed that RNase E is not involved in the maturation of all tRNAs (10,11,75–77) despite the presence of tandem arrangement of tRNAs or tRNA and transcriptional terminators. This suggest that other features are important determinants of cleavage, probably a combination of the spacing of structure elements, the accessibility of single-stranded regions and 5′-monophosphorylated ends, and the sequence of single-stranded regions accessible by the catalytic site. There are many other cases where individual features that are recognizable by RNase E are present on RNAs but appear to be insufficient to promote cleavage; for example, whilst it appears that many small regulatory RNAs deliver RNase E to specific sites in mRNA (73), there is a class of small RNAs that activate translation without initiating rapid RNA decay (78). A better understanding of the overall control of RNase E cleavage will almost certainly require atomistic descriptions of further RNase E–RNA complexes. Although progress is being make with computational protein–RNA docking, it is hampered by the inherent flexibility of RNA, the transient nature of many protein–RNA interactions and the propensity for global rearrangement upon binding (79).

## DATA AVAILABILITY

The data underlying this article are available in the article and in its online supplementary data.

## Supplementary Material

gkac1228_Supplemental_File

## References

[1] Wolf S.F. , Schlessinger D. Nuclear metabolism of ribosomal RNA in growing, methionine-limited, and ethionine-treated HeLa cells. Biochemistry. 1977; 16:2783–2791. 889788 10.1021/bi00631a031

[2] Calzone F.J. , Angerer R.C., Gorovsky M.A. Regulation of protein synthesis in *Tetrahymena*. Quantitative estimates of the parameters determining the rates of protein synthesis in growing, starved, and starved-deciliated cells. J. Biol. Chem. 1983; 258:6887–6898. 6853508

[3] Warner J.R. The economics of ribosome biosynthesis in yeast. Trends Biochem. Sci. 1999; 24:437–440. 10542411 10.1016/s0968-0004(99)01460-7

[4] Dong H. , Nilsson L., Kurland C.G. Co-variation of tRNA abundance and codon usage in *Escherichia coli* at different growth rates. J. Mol. Biol. 1996; 260:649–663. 8709146 10.1006/jmbi.1996.0428

[5] Bremer H. , Dennis P.P. Modulation of chemical composition and other parameters of the cell by growth rate. EcolSal Plus. 1987; 3:10.1128/ecosal.5.2.3. 26443740

[6] Deutscher M.P. Degradation of stable RNA in bacteria. J. Biol. Chem. 2003; 278:45041–45044. 12941949 10.1074/jbc.R300031200

[7] Shepherd J. , Ibba M. Bacterial transfer RNAs. FEMS Microbiol. Rev. 2015; 39:280–300. 25796611 10.1093/femsre/fuv004 PMC4542688

[8] Mackie G.A. RNase E: at the interface of bacterial RNA processing and decay. Nat. Rev. Microbiol. 2013; 11:45–57. 23241849 10.1038/nrmicro2930

[9] Li Z. , Deutscher M.P. Maturation pathways for *E. coli* tRNA precursors: a random multienzyme process *in vivo*. Cell. 1996; 86:503–512. 8756732 10.1016/s0092-8674(00)80123-3

[10] Li Z. , Deutscher M.P. RNase E plays an essential role in the maturation of *Escherichia coli* tRNA precursors. RNA. 2002; 8:97–109. 11871663 10.1017/s1355838202014929 PMC1370232

[11] Ow M.C. , Kushner S.R. Initiation of tRNA maturation by RNase E is essential for cell viability in *E. coli*. Genes Dev. 2002; 16:1102–1115. 12000793 10.1101/gad.983502 PMC186257

[12] Mohanty B.K. , Kushner S.R. Rho-independent transcription terminators inhibit RNase P processing of the *secG*, *leuU* and *metT* tRNA polycistronic transcripts in *Escherichia coli*. Nucleic Acids Res. 2008; 36:364–375. 18033800 10.1093/nar/gkm991 PMC2241853

[13] Clarke J.E. , Kime L., Romero A.,D., McDowall K.J. Direct entry by RNase E is a major pathway for the degradation and processing of RNA in *Escherichia coli*. Nucleic Acids Res. 2014; 42:11733–11751. 25237058 10.1093/nar/gku808 PMC4191395

[14] Kime L. , Clarke J.E., Romero A.,D., Grasby J.A., McDowall K.J. Adjacent single-stranded regions mediate processing of tRNA precursors by RNase E direct entry. Nucleic Acids Res. 2014; 42:4577–4589. 24452799 10.1093/nar/gkt1403 PMC3985628

[15] Swerdlow H. , Guthrie C. Structure of intron-containing tRNA precursors. Analysis of solution conformation using chemical and enzymatic probes. J. Biol. Chem. 1984; 259:5197–5207. 6371001

[16] Lan P. , Tan M., Zhang Y., Niu S., Chen J., Shi S., Qiu S., Wang X., Peng X., Cai G. et al. Structural insight into precursor tRNA processing by yeast ribonuclease P. Science. 2018; 362:eaat6678. 30262633 10.1126/science.aat6678

[17] Stead M.B. , Marshburn S., Mohanty B.K., Mitra J., Castillo L.P., Ray D., van Bakel H., Hughes T.R., Kushner S.R. Analysis of *Escherichia coli* RNase E and RNase III activity *in vivo* using tiling microarrays. Nucleic Acids Res. 2011; 39:3188–3203. 21149258 10.1093/nar/gkq1242 PMC3082872

[18] Hui M.P. , Foley P.L., Belasco J.G. Messenger RNA degradation in bacterial cells. Annu. Rev. Genet. 2014; 48:537–559. 25292357 10.1146/annurev-genet-120213-092340 PMC4431577

[19] Mackie G.A. Ribonuclease E is a 5′-end-dependent endonuclease. Nature. 1998; 395:720–723. 9790196 10.1038/27246

[20] Mackie G.A. Stabilization of circular *rpsT* mRNA demonstrates the 5′-end dependence of RNase E action *in vivo*. J. Biol. Chem. 2000; 275:25069–25072. 10871599 10.1074/jbc.C000363200

[21] Celesnik H. , Deana A., Belasco J.G. Initiation of RNA decay in *Escherichia coli* by 5′-pyrophosphate removal. Mol. Cell. 2007; 27:79–90. 17612492 10.1016/j.molcel.2007.05.038 PMC2196405

[22] Deana A. , Celesnik H., Belasco J.G. The bacterial enzyme RppH triggers messenger RNA degradation by 5′-pyrophosphate removal. Nature. 2008; 451:355–358. 18202662 10.1038/nature06475

[23] Luciano D.J. , Vasilyev N., Richards J., Serganov A., Belasco J.G. A novel RNA phosphorylation state enables 5′ end-dependent degradation in *Escherichia coli*. Mol. Cell. 2017; 67:44–54. 28673541 10.1016/j.molcel.2017.05.035 PMC5542582

[24] Anupama K. , Leela J.K., Gowrishankar J. Two pathways for RNase E action in *Escherichia coli in vivo* and bypass of its essentiality in mutants defective for Rho-dependent transcription termination. Mol. Microbiol. 2011; 82:1330–1348. 22026368 10.1111/j.1365-2958.2011.07895.x

[25] Kime L. , Jourdan S.S., Stead J.A., Hidalgo-Sastre A., McDowall K.J. Rapid cleavage of RNA by RNase E in the absence of 5′-monophosphate stimulation. Mol. Microbiol. 2010; 76:590–604. 19889093 10.1111/j.1365-2958.2009.06935.x PMC2948425

[26] Hankins J.S. , Zappavigna C., Prud’homme-Généreux A., Mackie G.A. Role of RNA structure and susceptibility to RNase E in regulation of a cold shock mRNA, *cspA* mRNA. J. Bacteriol. 2007; 189:4353–4358. 17416651 10.1128/JB.00193-07 PMC1913359

[27] Hajnsdorf E. , Braun F., Haugel-Nielsen J., le Derout J., Régnier P. Multiple degradation pathways of the *rpsO* mRNA of *Escherichia coli*: RNase E interacts with the 5′ and 3′ extremities of the primary transcript. Biochimie. 1996; 78:416–424. 8915531 10.1016/0300-9084(96)84748-1

[28] Braun F. , Hajnsdorf E., Régnier P. Polynucleotide phosphorylase is required for the rapid degradation of the RNase E-processed *rpsO* mRNA of *Escherichia coli* devoid of its 3′ hairpin. Mol. Microbiol. 1996; 19:997–1005. 8830280 10.1046/j.1365-2958.1996.440971.x

[29] Braun F. , le Derout J., Régnier P. Ribosomes inhibit an RNase E cleavage which induces the decay of the *rpsO* mRNA of *Escherichia coli*. EMBO J. 1998; 17:4790–4797. 9707438 10.1093/emboj/17.16.4790 PMC1170808

[30] Vytvytska O. , Moll I., Kaberdin V.R., von Gabain A., Bläsi U. Hfq (HF1) stimulates *ompA* mRNA decay by interfering with ribosome binding. Genes Dev. 2000; 14:1109–1118. 10809669 PMC316587

[31] Afonyushkin T. , Večerek B., Moll I., Bläsi U., Kaberdin V.R. Both RNase E and RNase III control the stability of *sodB* mRNA upon translational inhibition by the small regulatory RNA RyhB. Nucleic Acids Res. 2005; 33:1678–1689. 15781494 10.1093/nar/gki313 PMC1069011

[32] Baker K.E. , Mackie G.A. Ectopic RNase E sites promote bypass of 5′-end-dependent mRNA decay in *Escherichia coli*. Mol. Microbiol. 2003; 47:75–88. 12492855 10.1046/j.1365-2958.2003.03292.x

[33] McDowall K.J. , Cohen S.N. The N-terminal domain of the *rne* gene product has RNase E activity and is non-overlapping with the arginine-rich RNA-binding site. J. Mol. Biol. 1996; 255:349–355. 8568879 10.1006/jmbi.1996.0027

[34] Callaghan A.J. , Marcaida M.J., Stead J.A., McDowall K.J., Scott W.G., Luisi B.F. Structure of *Escherichia coli* RNase E catalytic domain and implications for RNA turnover. Nature. 2005; 437:1187–1191. 16237448 10.1038/nature04084

[35] Callaghan A.J. , Grossmann J.G., Redko Y.U., Ilag L.L., Moncrieffe M.C., Symmons M.F., Robinson C.V., McDowall K.J., Luisi B.F. Quaternary structure and catalytic activity of the *Escherichia coli* ribonuclease E amino-terminal catalytic domain. Biochemistry. 2003; 42:13848–13855. 14636052 10.1021/bi0351099

[36] Jourdan S.S. , Kime L., McDowall K.J. The sequence of sites recognised by a member of the RNase E/G family can control the maximal rate of cleavage, while a 5′-monophosphorylated end appears to function cooperatively in mediating RNA binding. Biochem. Biophys. Res. Commun. 2010; 391:879–883. 19945430 10.1016/j.bbrc.2009.11.156

[37] Jourdan S.S. , McDowall K.J. Sensing of 5′ monophosphate by *Escherichia coli* RNase G can significantly enhance association with RNA and stimulate the decay of functional mRNA transcripts *in vivo*. Mol. Microbiol. 2008; 67:102–115. 18078441 10.1111/j.1365-2958.2007.06028.x

[38] Bandyra K.J. , Wandzik J.M., Luisi B.F. Substrate recognition and autoinhibition in the central ribonuclease RNase E. Mol. Cell. 2018; 72:275–285. 30270108 10.1016/j.molcel.2018.08.039 PMC6202311

[39] Koslover D.J. , Callaghan A.J., Marcaida M.J., Garman E.F., Martick M., Scott W.G., Luisi B.F. The crystal structure of the *Escherichia coli* RNase E apoprotein and a mechanism for RNA degradation. Structure. 2008; 16:1238–1244. 18682225 10.1016/j.str.2008.04.017 PMC2631609

[40] Fang P. , Wang J., Li X., Guo M., Xing L., Cao X., Zhu Y., Gao Y., Niu L., Teng M. Crystallization and preliminary X-ray analysis of *Escherichia coli* RNase G. Acta Crystallogr. Sect. F Struct. Biol. Cryst. Commun. 2009; 65:586–588. 10.1107/S1744309109015802 PMC2688416 19478437

[41] Wachi M. , Umitsuki G., Nagai K. Functional relationship between *Escherichia coli* RNase E and the CafA protein. Mol. Gen. Genet. 1997; 253:515–519. 9037114 10.1007/s004380050352

[42] McDowall K.J. , Kaberdin V.R., Wu S.W., Cohen S.N., Lin-Chao S. Site-specific RNase E cleavage of oligonucleotides and inhibition by stem–loops. Nature. 1995; 374:287–290. 7533896 10.1038/374287a0

[43] Schneider C.A. , Rasband W.S., Eliceiri K.W. NIH Image to ImageJ: 25 years of image analysis. Nat. Methods. 2012; 9:671–675. 22930834 10.1038/nmeth.2089 PMC5554542

[44] Seifert E. OriginPro 9.1: scientific data analysis and graphing software—software review. J. Chem. Inf. Model. 2014; 54:1552. 24702057 10.1021/ci500161d

[45] Jiang X. , Diwa A., Belasco J.G. Regions of RNase E important for 5′-end-dependent RNA cleavage and autoregulated synthesis. J. Bacteriol. 2000; 182:2468–2475. 10762247 10.1128/jb.182.9.2468-2475.2000 PMC111309

[46] Jain C. , Belasco J.G. RNase E autoregulates its synthesis by controlling the degradation rate of its own mRNA in *Escherichia coli*: unusual sensitivity of the *rne* transcript to RNase E activity. Genes Dev. 1995; 9:84–96. 7530223 10.1101/gad.9.1.84

[47] Jiang X. , Belasco J.G. Catalytic activation of multimeric RNase E and RNase G by 5′-monophosphorylated RNA. Proc. Natl Acad. Sci. USA. 2004; 101:9211–9216. 15197283 10.1073/pnas.0401382101 PMC438955

[48] Garrey S.M. , Mackie G.A. Roles of the 5′-phosphate sensor domain in RNase E. Mol. Microbiol. 2011; 80:1613–1624. 21518390 10.1111/j.1365-2958.2011.07670.x

[49] Jones B.N. , Quang-Dang D.U., Oku Y., Gross J.D. A kinetic assay to monitor RNA decapping under single-turnover conditions. Methods Enzymol. 2008; 448:23–40. 19111169 10.1016/S0076-6879(08)02602-5

[50] Redko Y. , Tock M.R., Adams C.J., Kaberdin V.R., Grasby J.A., McDowall K.J. Determination of the catalytic parameters of the N-terminal half of *Escherichia coli* ribonuclease E and the identification of critical functional groups in RNA substrates. J. Biol. Chem. 2003; 278:44001–44008. 12947103 10.1074/jbc.M306760200

[51] Himabindu P. , Anupama K. Decreased expression of stable RNA can alleviate the lethality associated with RNase E deficiency in *Escherichia coli*. J. Bacteriol. 2017; 199:e00724-16. 28167522 10.1128/JB.00724-16 PMC5370413

[52] Lopez P.J. , Marchand I., Joyce S.A., Dreyfus M. The C-terminal half of RNase E, which organizes the *Escherichia coli* degradosome, participates in mRNA degradation but not rRNA processing *in vivo*. Mol. Microbiol. 1999; 33:188–199. 10411735 10.1046/j.1365-2958.1999.01465.x

[53] Leroy A. , Vanzo N.F., Sousa S., Dreyfus M., Carpousis A.J. Function in *Escherichia coli* of the non-catalytic part of RNase E: role in the degradation of ribosome-free mRNA. Mol. Microbiol. 2002; 45:1231–1243. 12207692 10.1046/j.1365-2958.2002.03104.x

[54] Ow M.C. , Perwez T., Kushner S.R. RNase G of *Escherichia coli* exhibits only limited functional overlap with its essential homologue, RNase E. Mol. Microbiol. 2003; 49:607–622. 12864847 10.1046/j.1365-2958.2003.03587.x

[55] Lee K. , Bernstein J.A., Cohen S.N. RNase G complementation of *rne* null mutation identifies functional interrelationships with RNase E in *Escherichia coli*. Mol. Microbiol. 2002; 43:1445–1456. 11952897 10.1046/j.1365-2958.2002.02848.x

[56] Chung D.H. , Min Z., Wang B.C., Kushner S.R. Single amino acid changes in the predicted RNase H domain of *Escherichia coli* RNase G lead to complementation of RNase E deletion mutants. RNA. 2010; 16:1371–1385. 20507976 10.1261/rna.2104810 PMC2885686

[57] Garrey S.M. , Blech M., Riffell J.L., Hankins J.S., Stickney L.M., Diver M., Hsu Y.H.R., Kunanithy V., Mackie G.A. Substrate binding and active site residues in RNases E and G: role of the 5′-sensor. J. Biol. Chem. 2009; 284:31843–31850. 19778900 10.1074/jbc.M109.063263 PMC2797255

[58] Callaghan A.J. , Redko Y., Murphy L.M., Grossmann J.G., Yates D., Garman E., Ilag L.L., Robinson C.v., Symmons M.F., McDowall K.J. et al. Zn-Link’: a metal-sharing interface that organizes the quaternary structure and catalytic site of the endoribonuclease, RNase E. Biochemistry. 2005; 44:4667–4675. 15779893 10.1021/bi0478244

[59] Deana A. , Belasco J.G. The function of RNase G in *Escherichia coli* is constrained by its amino and carboxyl termini. Mol. Microbiol. 2004; 51:1205–1217. 14763991 10.1046/j.1365-2958.2003.03905.x

[60] Updegrove T.B. , Kouse A.B., Bandyra K.J., Storz G. Stem–loops direct precise processing of 3′ UTR-derived small RNA MicL. Nucleic Acids Res. 2019; 47:1482–1492. 30462307 10.1093/nar/gky1175 PMC6379649

[61] Ehretsmann C.P. , Carpousis A.J., Krisch H.M. Specificity of *Escherichia coli* endoribonuclease RNase E: *i**n vivo* and *in vitro* analysis of mutants in a bacteriophage T4 mRNA processing site. Genes Dev. 1992; 6:149–159. 1730408 10.1101/gad.6.1.149

[62] Cormack R.S. , Mackie G.A. Structural requirements for the processing of *Escherichia coli* 5S ribosomal RNA by RNase E *in vitro*. J. Mol. Biol. 1992; 228:1078–1090. 1474579 10.1016/0022-2836(92)90316-c

[63] Diwa A.A. , Belasco J.G. Critical features of a conserved RNA stem–loop important for feedback regulation of RNase E synthesis. J. Biol. Chem. 2002; 277:20415–20422. 11919204 10.1074/jbc.M202313200

[64] Bardey V. , Vallet C., Robas N., Charpentier B., Thouvenot B., Mougin A., Hajnsdorf E., Régnier P., Springer M., Branlant C. Characterization of the molecular mechanisms involved in the differential production of erythrose-4-phosphate dehydrogenase, 3-phosphoglycerate kinase and class II fructose-1,6-bisphosphate aldolase in *Escherichia coli*. Mol. Microbiol. 2005; 57:1265–1287. 16102000 10.1111/j.1365-2958.2005.04762.x

[65] Jeon H.J. , Kang C., Monford Paul Abishek N., Lee Y., Wang X., Chattoraj D.K., Lim H.M. Translation initiation control of RNase E-mediated decay of polycistronic *gal* mRNA. Front. Mol. Biosci. 2020; 7:586413. 33240931 10.3389/fmolb.2020.586413 PMC7681074

[66] Alifano P. , Rivellini F., Piscitelli C., Arraiano C.M., Bruni C.B., Carlomagno M.S. Ribonuclease E provides substrates for ribonuclease P-dependent processing of a polycistronic mRNA. Genes Dev. 1994; 8:3021–3031. 8001821 10.1101/gad.8.24.3021

[67] Mackie G.A. Determinants in the *rpsT* mRNAs recognized by the 5′-sensor domain of RNase E. Mol. Microbiol. 2013; 89:388–402. 23734704 10.1111/mmi.12283

[68] Dar D. , Sorek R. High-resolution RNA 3′-ends mapping of bacterial Rho-dependent transcripts. Nucleic Acids Res. 2018; 46:6797–6805. 29669055 10.1093/nar/gky274 PMC6061677

[69] Hajnsdorf E. , Braun F., Haugel-Nielsen J., Régnier P. Polyadenylylation destabilizes the *rpsO* mRNA of *Escherichia coli*. Proc. Natl Acad. Sci. USA. 1995; 92:3973–3977. 7732015 10.1073/pnas.92.9.3973 PMC42084

[70] Marujo P.E. , Hajnsdorf E., le Derout J., Andrade R., Arraiano C.M., Régnier P. RNase II removes the oligo(A) tails that destabilize the *rpsO* mRNA of *Escherichia coli*. RNA. 2000; 6:1185–1193. 10943897 10.1017/s135583820000073x PMC1369992

[71] Andrade J.M. , Pobre V., Silva I.J., Domingues S., Arraiano C.M. The role of 3′–5′ exoribonucleases in RNA degradation. Prog. Mol. Biol. Transl. Sci. 2009; 85:187–229. 19215773 10.1016/S0079-6603(08)00805-2

[72] Irastortza-Olaziregi M. , Amster-Choder O. Coupled transcription–translation in prokaryotes: an old couple with new surprises. Front. Microbiol. 2021; 11:624830. 33552035 10.3389/fmicb.2020.624830 PMC7858274

[73] Waters S.A. , McAteer S.P., Kudla G., Pang I., Deshpande N.P., Amos T.G., Leong K.W., Wilkins M.R., Strugnell R., Gally D.L. et al. Small RNA interactome of pathogenic *E. coli* revealed through crosslinking of RNase E. EMBO J. 2017; 36:374–387. 27836995 10.15252/embj.201694639 PMC5286369

[74] Schuck A. , Diwa A., Belasco J.G. RNase E autoregulates its synthesis in *Escherichia coli* by binding directly to a stem–loop in the *rne* 5′ untranslated region. Mol. Microbiol. 2009; 72:470–478. 19320830 10.1111/j.1365-2958.2009.06662.x PMC2857391

[75] Mohanty B.K. , Kushner S.R. Ribonuclease P processes polycistronic tRNA transcripts in *Escherichia coli* independent of ribonuclease E. Nucleic Acids Res. 2007; 35:7614–7625. 17981836 10.1093/nar/gkm917 PMC2190699

[76] Mohanty B.K. , Petree J.R., Kushner S.R. Endonucleolytic cleavages by RNase E generate the mature 3′ termini of the three proline tRNAs in *Escherichia coli*. Nucleic Acids Res. 2016; 44:6350–6362. 27288443 10.1093/nar/gkw517 PMC5291269

[77] Agrawal A. , Mohanty B.K., Kushner S.R. Processing of the seven valine tRNAs in *Escherichia coli* involves novel features of RNase P. Nucleic Acids Res. 2014; 42:11166–11179. 25183518 10.1093/nar/gku758 PMC4176162

[78] Papenfort K. , Vanderpool C.K. Target activation by regulatory RNAs in bacteria. FEMS Microbiol. Rev. 2015; 39:362–378. 25934124 10.1093/femsre/fuv016 PMC4542691

[79] Fulle S. , Gohlke H. Molecular recognition of RNA: challenges for modelling interactions and plasticity. J. Mol. Recognit. 2010; 23:220–231. 19941322 10.1002/jmr.1000

