# Supplementary material for: The recognition of structured elements by a conserved groove distant from domains associated with catalysis is an essential determinant of RNase E
Source: Nucleic Acids Res. 2023 Jan 3;51(1):365–79. doi: 10.1093/nar/gkac1228 (PMC9841416; doi:10.1093/nar/gkac1228)
Supplement: gkac1228_Supplemental_File [file gkac1228_supplemental_file.docx]

**Supplementary Material**

**
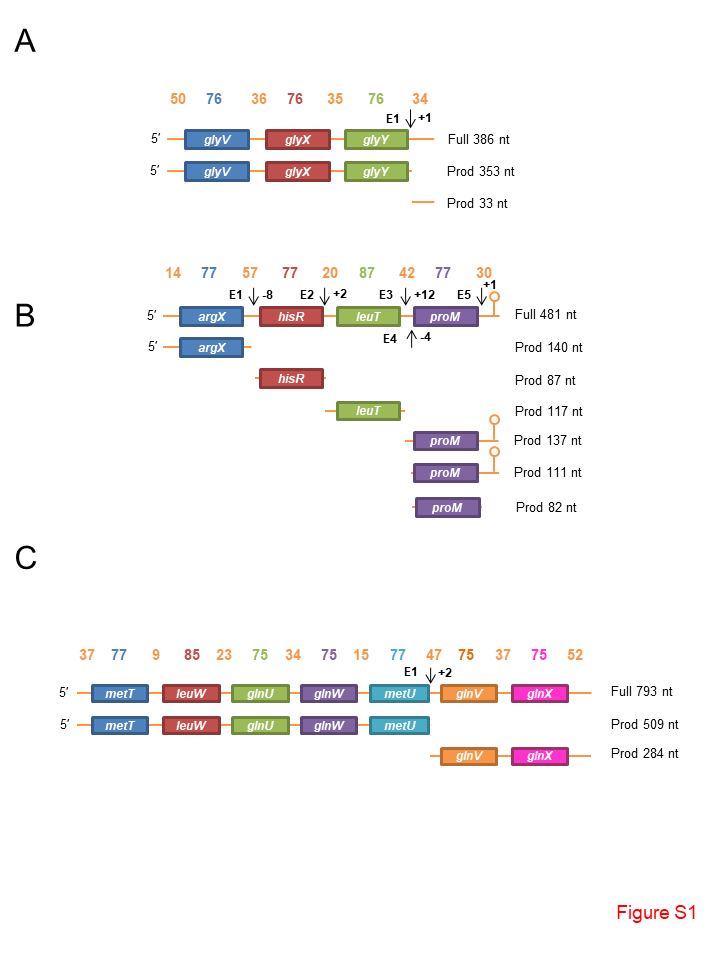
**

**Figure S1. Schematic of tRNA precursors from *E. coli* that are processed by RNase E.** Panel A shows a schematic of the *glyV-glyX-glyY* tRNA precursor. The tRNA units are labelled and coloured, with coloured numbers represent the length in nt of each unit of the full transcript. The orange numbers represent the length in nucleotides of the corresponding intergenic regions. Vertical arrows show the position of an RNase E site(s), with the number to the right of the arrow corresponding to the exact position relative to the start or end of the nearest tRNA unit (“+” is downstream and “-” is upstream). Below each full-length schematic is shown a schematic of the upstream and downstream products generated upon RNase E cleavage. The length in nt of the full-length transcript and the upstream and downstream products of RNase E cleavage are shown to the right. Panel B shows a schematic of the *argX-hisR-leuT-proM* tRNA precursor. Labelling is as Panel A. A Rho-independent transcription terminator is also shown. Panel C shows a schematic of the *metT-leuW-glnU-glnW-metU-glnV-glnX* tRNA precusor. Labelling is as Panel A.

**
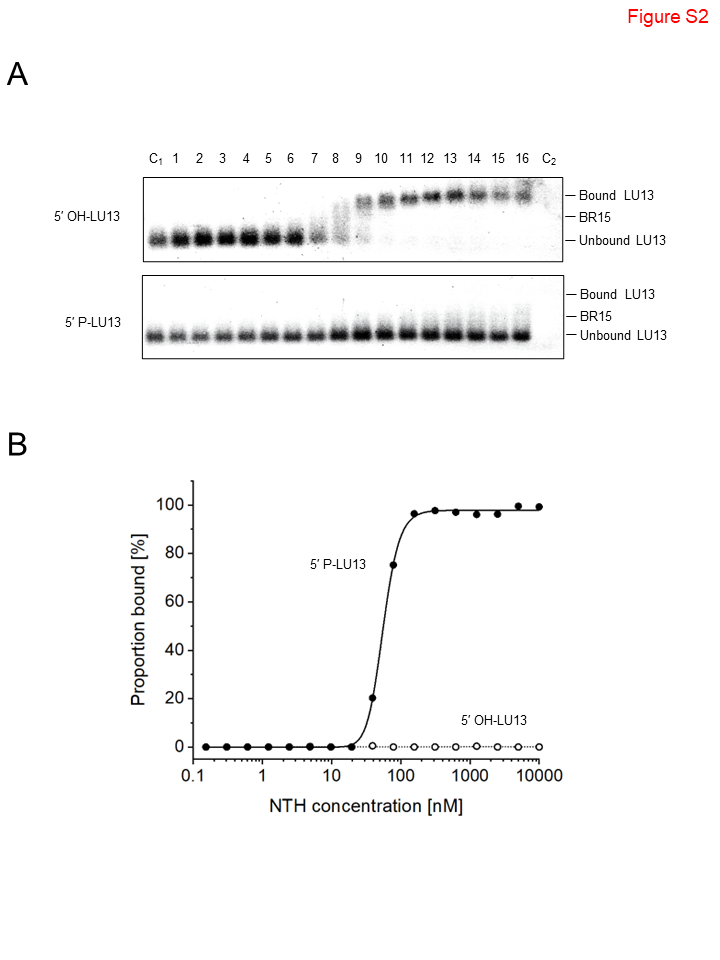
**

**Figure S2. Binding of RNase E to the standard substrate LU13.** Panel A shows electrophoretic mobility shift assays (EMSAs) of LU13 RNA fragments incubated with increasing concentrations of NTH-RNase E with the D346N substitution. Lanes 1-14 contain 20 nM RNA fragments incubated with 0.3, 0.6, 1.2, 2.4, 4.9, 9.8, 19.5, 39, 78.1, 156.3, 312.5, 625 nM, 1.25, 2.5, 5 and 10 µM NTH-RNase E D346N, respectively. Lanes C_1_ and C_2_ contain 20 nM RNA fragments and 2.5 µM NTH-RNase E D346N, respectively. The first and second images (from top to bottom) correspond to LU13 with 5′-monophosphorylated and 5′-hydroxylated ends, respectively. Panel B shows binding curves of this data. The closed and open circles corresponding to the 5′-monophosphorylated and 5′-hydroxylated form of LU13, respectively.


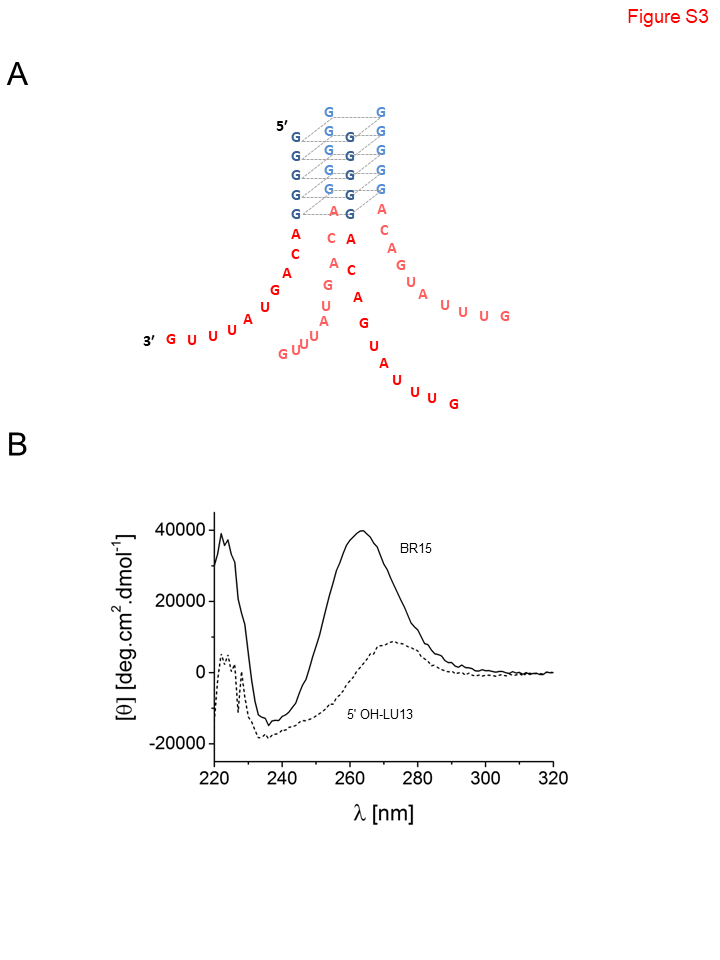


**Figure S3. Characterisation of BR15.** Panel A shows a molecular representation of the BR15 quadruplex. Sequences in blue and red show unmodified and 2′ *O-*methylated ribonucleotides, respectively. Panel B shows the circular dichroism spectrum of BR15 (open circles) compared to the control 5′-hydroxylated LU13 (closed circles), which lacks a string of G’s required for quadruplex formation. The LU13 and BR15 substrates were synthesised by MWG operon (Eurofins, Germany). Substrates were diluted to 7.5 µM in 25 mM *bis*-Tris Propane (pH 8.3), 100 mM KCl, 15 mM MgCl_2_, 0.1% (v/v) Triton X-100, 1 mM dithiothreitol and CD spectra were obtained at 37°C. Molar ellipticity ([θ]) was calculated at 5 nm wavelength (λ) intervals between 220 and 320 nm. High tension values remained below 400 mV throughout the spectra.


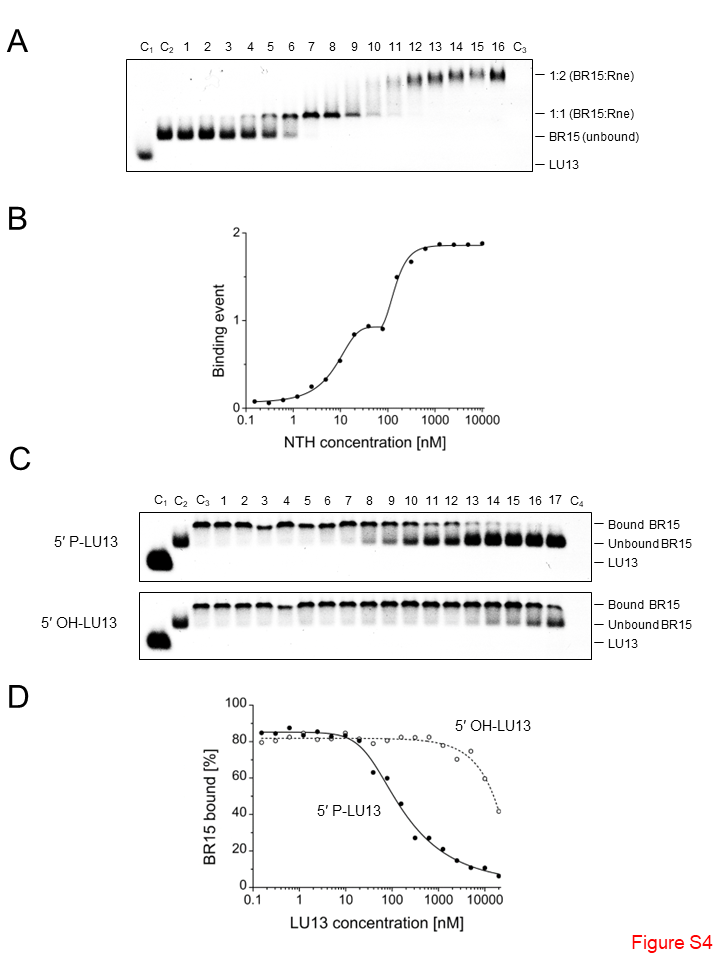


**Figure S4. Binding of RNase E to BR15 in the absence and presence of competitor RNA.** Panel A shows an electrophoretic mobility shift assay (EMSA) of labelled BR15 incubated with increasing concentrations of NTH-RNase E. Lanes 1 to 16 contain 7.5 nM BR15 (quadruplex) incubated with 0.3, 0.6, 1.2, 2.4, 4.9, 9.8, 19.5, 39, 78.1, 156.3, 312.5, 625 nM, 1.25, 2.5, 5, and 10 µM wild-type NTH-RNase E. Lanes C_1_, C_2_ and C_3_ contain 30 nM LU13, 7.5 nM BR15, and 10 µM wild-type NTH-RNase E, respectively. Labelling indicates the position of two resulting complexes, whose production is consistent with a model in which each dimeric unit of RNase E tetramer can bind a BR15 quadruplex. The expected ratios of BR15 quadruplex to RNase E tetramer in each of the complexes is indicated within brackets. Panel B shows a binding curve of BR15 to NTH-RNase E. The proportion of BR15 in each binding state was calculated from the band intensities by 2D densitometry and plotted as a function of NTH-RNase E concentration. The *K*_d_ values for the formation of each complex, as determined by fitting a 1:1 binding model separately to each of the two phases of binding, were 7.8 and 139 nM. The higher *K*_d_ value for the second interaction is presumably due to spatial constraints imposed by the first. Panel C shows competition between unlabelled LU13 and labelled BR15 in a complex with NTH-RNase E. Lanes 1 to 17 contain 7.5 nM BR15 and 20 nM NTH-RNase E incubated with 0.3, 0.6, 1.2, 2.4, 4.9, 9.8, 19.5, 39, 78.1, 156.3, 312.5, 625 nM, 1.25, 2.5, 5, 10, and 20 µM LU13. Lanes C_1_, C_2_, C_3_ and C_4_ contain 30 nM LU13 with a 3’-fluorescein label, 7.5 nM BR15, 7.5 nM BR15 incubated with 20 nM wild-type NTH-RNase E, and 20 nM NTH-RNase E incubated with 20 µM unlabelled LU13. The top and bottom images in this panel correspond to 5′-monophosphorylated and 5′-hydroxylated LU13, respectively. Panel D shows the proportion of labelled BR15 bound to NTH-RNase E in the presence of increasing amounts of unlabelled LU13, as determined by 2D densitometry. The closed and open circles corresponding to the 5′-monophosphorylated and 5′-hydroxylated form of LU13, respectively.


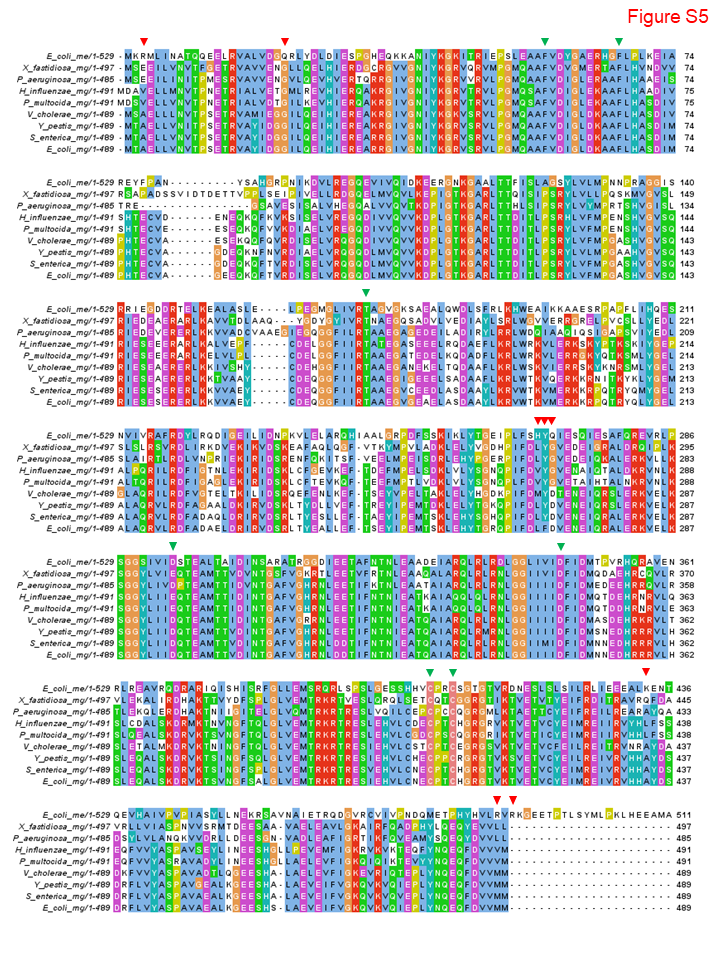


**Figure S5. Amino acid composition of RNase G paralogues and RNase E**. A Multiple-sequence alignment was performed with *E. coli* K12 RNase E and RNase G from various bacterial species using ClustalW. Data was visualised on Jalview. Chemically conserved residues between 4 or more queries are highlighted with coloured boxes. Residues that have been described previously to be conserved between RNase E and G are highlighted with green arrows. Residues that are associated with the RNA-binding groove are highlighted with red arrows.

| **Name** | **Description** | **Sequence (5’ to 3’)** |
| --- | --- | --- |
| *g*386 Forward  *g*224 Forward  *g*148 Forward  *g*386 Reverse  *a*225 Forward  *a*225 Reverse  *a*148 Reverse  *m*793 Forward  *m*456 Forward  *m*381 Forward  *m*793 Reverse  *m*329 Reverse  *m*292 Reverse  *m*217 Reverse  *glnW* Reverse  *hisR*D1 Reverse  *hisR*D2 Reverse  *hisR*D3 Reverse  *argXrpsO* Reverse | *g*386 cDNA  *g*224 cDNA  *g*148 cDNA  *g*386, 224, 148 cDNA  *a*225, 148, *hisR* arm deletions cDNA  *a*225 cDNA  *a*148 cDNA  *m*793 cDNA  *m*456, 329, 292, 217, *glnW* cDNA  *m*381 and 3^rd^ fragment (Fig 2A) cDNA  *m*793, 456, 381 cDNA  *m*329 cDNA  *m*292 cDNA  *m*217 and 3^rd^ fragment (Fig 2A) cDNA  *glnW* cDNA  Removal of T arm of *hisR*  Removal of D arm of *hisR*  Removal of T and D arm of *hisR*  Replacement of *hisR* with *rpsO* terminator | ATCCTAATACGACTCACTATAGGGCCGTAACGACGCAGAAATG  ATCCTAATACGACTCACTATAGGGGCGGGAATAGCTCAGTTGG  ATCCTAATACGACTCACTATAGGGAAATTTGAAAGTGCTGTAAGGCA  GCGTCGCTGTGGATATTTTATT  ATCCTAATACGACTCACTATAGGGAACGGCGCTAAGCGCCCG  TGGGGTGGCTAATGGGATT  ACTACAAATCTTGTTACGCGGT  ATCCTAATACGACTCACTATAGGGCGCAACGCCGATAAGGTA  ATCCTAATACGACTCACTATAGGGTGGGGTATCGCCAAGC  ATCCTAATACGACTCACTATAGGGTCGAAGAAACAATCTGGCTAC  ATTGAATGAACGCAGAAAAGC  ACTGGGTGCACTTACAAGGTA  TGGCTGGGGTACGAGGAT  ACAAATTGGTTTTGAATTTGCC  TGGCTGGGGTACCTGGAT  **TGGGGTGGCTAGACAACTGGAATCACAATCCAGGGCTCTACCAACTGAGCTATAGCCACC**ACTACAAATCTTGTTACGCG  **TGGGGTGGCTAATGGGATTCGAACCCACGACAACTGGAATCACAATCCAGGTATAGCCACC**ACTACAAATCTTGTTACGCG  **TGGGGTGGCTAGACAACTGGAATCACAATCCAGGTATAGCCACC**ACTACAAATCTTGTTACGCG  **TTGAAAAAAGGGGCCACTCAGGCCCCCTTTTC**ACTACAAATCTTGTTACGCG |

**Table S1 DNA primers for PCR generation of cDNA templates.** Underlined shows the T7 promoter to permit *in vitro* T7 transcription of RNA fragments. As a consequence, a GGG is present at the 5’ end of every RNA fragment. In bold shows the overhangs that contain the modified *hisR* region with T and/or D arm deletions, or the *rpsO* Rho-independent transcriptional terminator.

| Name | Sequence (5’ to 3’) |
| --- | --- |
| R3Q Forward | ctgctgagttgcgttgattaacat**ctg**tttcatatgacgaccttcgatatgg |
| R3Q Reverse | ccatatcgaaggtcgtcatatgaaa**cag**atgttaatcaacgcaactcagcag |
| Q22N Forward | Atccaggtcatacagacg**a**t**t**cccatctacaagggcaac |
| Q22N Reverse | Gttgcccttgtagatggg**a**a**t**cgtctgtatgacctggat |
| HYQ268269270SFN Forward | ggactcgatctgtgactcgat**a**t**t**g**a**ag**ct**gctgaacagcgggatctcgcc |
| HYQ268269270SFN Reverse | ggcgagatcccgctgttcagc**ag**ct**t**c**a**a**t**atcgagtcacagatcgagtcc |
| K433N Forward | cgaagaagaagcgctgaa**t**gagaacacccagg |
| K433N Reverse | cctgggtgttctc**a**ttcagcgcttcttcttcg |
| R488490Q Forward | GGGGTTTCTTCCCCTTT**CT**GCAC**CT**GCAGCACGTGGTAGTGC |
| R488490Q Reverse | GCACTACCACGTGCTGC**AG**GTGC**AG**AAAGGGGAAGAAACCCC |
| R3Q_pRNE1000 Forward | GTTGCCCTTGTAGATGGG**A**A**T**CGTCTGTATGACCTGGAT |
| R3Q_pRNE1000 Reverse | ATCCAGGTCATACAGACG**A**T**T**CCCATCTACAAGGGCAAC |
| Q22N_pRNE1000 Forward | GATCTGAATGGGGCCGCAAGATCTAAA**CAG**ATGTTAATCAACGCAACTCAG |
| Q22N_pRNE1000 Reverse | CTGAGTTGCGTTGATTAACAT**CTG**TTTAGATCTTGCGGCCCCATTCAGATC |
| 529STOP_pRNE1000 Forward | TCCGGCATGGCAAAGGT**CTA**CAGCGCAGGTTGTTCCG |
| 529STOP_pRNE1000 Reverse | CGGAACAACCTGCGCTG**TAG**ACCTTTGCCATGCCGGA |

**Table S2 DNA primers for site-directed mutagenesis.** In bold shows the point mutations introduced into the DNA sequence to confer the amino acid changes labelled.
